# Progress in Transparent Nano-Ceramics and Their Potential Applications

**DOI:** 10.3390/nano12091491

**Published:** 2022-04-27

**Authors:** Wuyi Ming, Zhiwen Jiang, Guofu Luo, Yingjie Xu, Wenbin He, Zhuobin Xie, Dili Shen, Liwei Li

**Affiliations:** 1 Henan Key Lab of Intelligent Manufacturing of Mechanical Equipment, Zhengzhou University of Light Industry, Zhengzhou 450002, China; mingwuyi@gmail.com (W.M.); jzw336699@163.com (Z.J.); xyj15203873137@163.com (Y.X.); hwb@zzuli.edu.cn (W.H.); liliwei@zzuli.edu.cn (L.L.); 2 Guangdong HUST Industrial Technology Research Institute, Guangdong Provincial Key Laboratory of Digital Manufacturing Equipment, Dongguan 523808, China; xzb18336947581@163.com; 3 School of Mechanical-Electronic and Automobile Engineering, Zhengzhou Institute of Technology, Zhengzhou 450052, China; shendili@163.com

**Keywords:** transparent nano-ceramics, nano powder, microstructure, optical transmittance, IR transmittance, magneto-optical material, mechanical strength, preparation method

## Abstract

Transparent nano-ceramics have an important high-transmittance, material-integrating structure and function and a variety of potential applications, such as use in infrared windows, optical isolators, composite armors, intelligent terminal screens, and key materials of solid-state lasers. Transparent ceramics were originally developed to replace single crystals because of their low fabricating cost, controllable shape, and variable composition. Therefore, this study reviews and summarizes the development trends in transparent nano-ceramics and their potential applications. First, we review the research progress and application of laser nano-ceramic materials, focusing on the influence of controllable doping of rare earth ions on thermal conductivity and the realization of large-scale fabrication technology. Second, the latest research progress on magneto-optical transparent nano-ceramics, mainly including terbium gallium garnet (Tb_3_Ga_5_O_12_, TGG) ceramics and terbium aluminum garnet (Tb_3_Al_5_O_12_, TAG) ceramics, are summarized, and their performance is compared. Third, the research progress of transparent armor nano-ceramic materials, represented by MgAl_2_O_3_ and Aluminum oxynitride (AlON), are reviewed. Lastly, the progress in electro-optical transparent nano-ceramics and scintillation transparent nano-ceramics is reported, and the influence of the material-fabrication process on electro-optic effect or luminous intensity is compared. Moreover, the effect of particle diameter on fabrication, the relationship between nano powder and performance, and different sintering methods are discussed. In summary, this study provides a meaningful reference for low-cost and sustainable production in the future.

## 1. Introduction

Transparent materials are essential for human beings. Advanced transparent materials have gradually replaced traditional materials, namely glass, for relevant technical applications where conventional materials are not applicable. Traditional transparent materials include glass, polymer, and alkali metal hydride, which have weak mechanical strength and unstable chemical properties [1]. Optical transparency may also be seen in single crystals of several inorganic materials. They are more stable and robust than typical materials. Single crystal growth, on the other hand, is frequently referred to as “growth” since it is primarily governed by rather slow thermodynamic processes. In addition, the single crystal with large growth is often challenging, especially for oxide materials with a high melting point, and the single crystal during growth cannot be used directly [2]. With the rapid development of nanomaterial technology, the preparation of high-purity nano powder can be realized [3,4,5]. The optimization of raw material powder promotes the densification process of transparent nano-ceramics, reduces the impurity content, and is a great breakthrough in the optical quality and various properties of ceramics.

**Figure 1:**
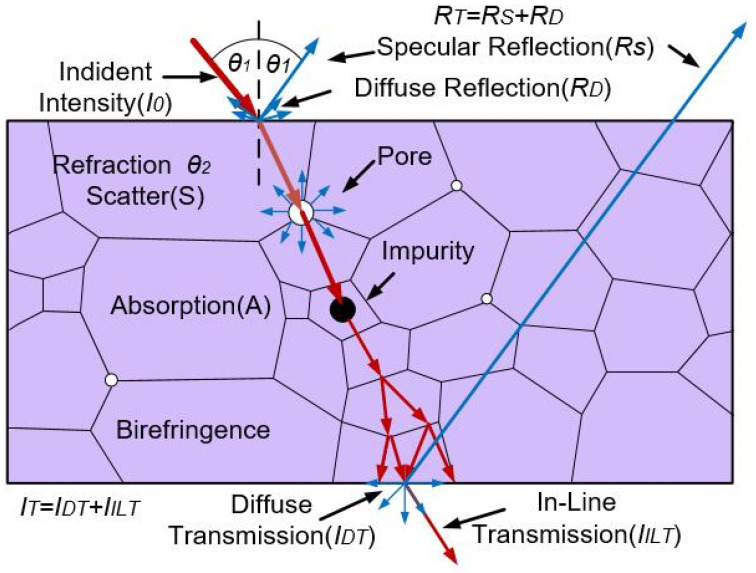
Sketch of light transmission in a polycrystalline ceramic.

Because ceramic is an inorganic polycrystalline material, it generally does not have transparency. This is because there are a lot of defects, such as pores and impurities in the ceramic, which cause scattering and refraction loss to the light incident into the ceramic so that the incident light cannot pass through the ceramic. Figure 1 demonstrates the schematic light transmission phenomena in a polycrystalline ceramic, which has more light scattering sources than a single crystal, such as grain boundaries, pores, impurities, and birefringence [6]. Initially, the incident intensity (*I*_0_) of light depends on the surface roughness and is composed of diffuse reflection (*R_D_*) and diffuse transmission (*I_DT_*). In addition, a certain amount of light is reflected on each surface of the material through specular reflection (*R_T_*). Light passing through polycrystalline materials can also interact with pores (residual pore scattering), grain boundaries (second phase scattering), impurities (inclusion absorption or scattering), and birefringence (birefringence of the non-cubic phase) [6]. The main factors affecting the optical quality of ceramics are light absorption, scattering of pores and the second phase, grain boundary reflection and refraction (birefringence), grain boundary scattering, and light scattering caused by surface roughness. Based on the above factors, the transmittance of ceramics can be calculated by Equation (1):

(1) $$\frac{I}{I_{0}}={\left(1-R\right)}^{2}e^{-mx}$$

where *I*_0_ is the incident intensity of light, *I* is the transmitted intensity of light, *m* is the light absorption coefficient, *x* is the sample thickness, and *R* is the light reflectivity [7]. The light absorption coefficient *m* can be given by Equation (2),

(2) $$m=\alpha +S_{im}+S_{op}$$

where α is the absorption coefficient of electron transition, $S_{im}$ is the scattering caused by structural heterogeneity (e.g., pores, second phase), and $S_{op}$ is the scattering caused by optical anisotropy (e.g., hexagonal system and other non-cubic systems).

According to Equations (1) and (2), the necessary conditions for preparing high-quality transparent nano-ceramics are [8]:

(1) High relative density and residual porosity less than 0.01%;
(2) No optical anisotropy;
(3) The grain boundary is free of impurities and second phase;
(4) The selective absorption of the crystal to the incident light is small;
(5) The surface is flat and has low roughness.

Therefore, polycrystalline transparent nano-ceramics with excellent optical properties can be fabricated by using high-purity raw material powder [9,10], especially the application of nano powder, reducing impurities and eliminating pores through certain processes, and reducing scattering caused by surface roughness through polishing and other means.

**Figure 2:**
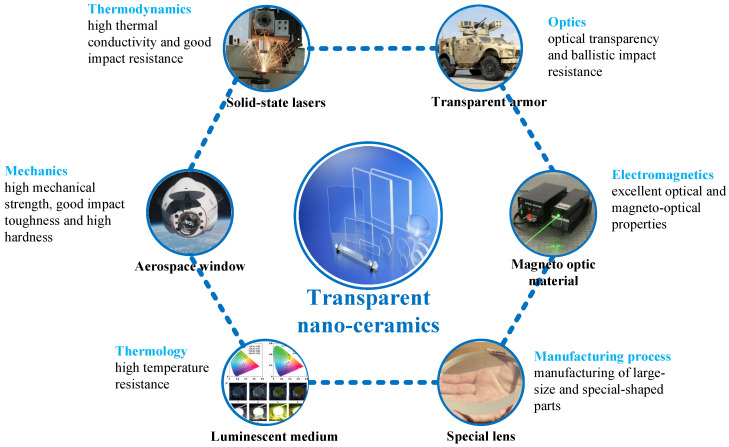
Performance requirements and applications of transparent nano-ceramics.

Compared with common optical function transparent single crystal or glass materials, transparent nano-ceramic materials have obvious advantages in their optical function effect, mechanical and thermal properties, low cost, and large size (compared with single crystal materials). At the same time, being ceramic materials, they have high strength and hardness, high-temperature resistance, and corrosion resistance, which are better than general optical materials [11]. With the improvement of the performance of transparent nano-ceramics, therefore, their functional applications have also been further developed. Research in many fields is gradually becoming commercialized, and the market potential is larger in the future. For example, transparent nano-ceramics have been used in laser gain media, transparent armor, aerospace windows, solid-state lighting, magneto-optic material, and other fields [2,11], as shown in Figure 2.

In this study, published literature was selected from databases such as Web of Science, Google Scholar, CNKI, and the Engineering Index, as well as publishers’ databases, such as Elsevier, IEEE Xplore, and Springer. A total of nearly 130 related documents were collected, and 100 documents are reviewed and cited in this study. This literature covers the applications of transparent nano-ceramics. Among them, Web of Science, IEEE Xplore, and other academic databases are rich in literature with a wide range of research. There are also many excellent Chinese journals and dissertations in the research results included by CNKI, so we also chose a small number of classic Chinese journals. Nano powder, microstructure, and preparation methods are all the key points that need to be considered in the study of transparent nano-ceramics. Therefore, when searching literature, keywords related to transparent nano-ceramics (such as “nano-ceramics”, “nano powder”, “microstructure”, and “preparation method”) and keywords related to material properties (such as “optical transmittance”, “IR transmittance”, “magneto-optical material”, and “mechanical strength”) were used. Categories and application areas of nanomaterials (e.g., “magneto-optical transparent nano-ceramics”, “armor transparent nano-ceramics”, “electro-optical transparent nano-ceramics”, and “scintillation transparent nano-ceramics”) were also indexed.

Then, this review summarizes the different applications of transparent nano-ceramics, mainly including laser transparent nano-ceramics, magneto-optical transparent nano-ceramics, transparent armor nano-ceramics, electro-optical transparent nano-ceramics, and scintillation transparent nano-ceramics. Section 2 reviews the research progress of transparent nano-ceramics in solid-state laser applications, focusing on the influence of the controllable doping of rare earth ions on their performance and the realization of large-scale fabrication technology. In Section 3, we summarize the latest research progress of magneto-optical transparent nano-ceramics, mainly including terbium gallium garnet (Tb_3_Ga_5_O_12_, TGG) transparent nano-ceramics and terbium aluminum garnet (Tb_3_Al_5_O_12_, TAG) transparent nano-ceramics, and we compare their properties and predict their future potential applications. Section 4 mainly reviews the research progress of transparent armor nano-ceramic materials, represented by MgAl_2_O_3_ and AlON. The potential applications of transparent armor nano-ceramic are aerospace windows, missile fairings, bulletproof windows, and other fields, which require high hardness and strength, good wear resistance, and impact resistance. In Section 5, we report the progress in electro-optical transparent nano-ceramics and scintillation transparent nano-ceramics. The influence of the material-fabrication process on the electro-optic effect or luminous intensity is compared. In Section 6, the advantages and disadvantages of the fabrication method of transparent nanoceramic material are discussed. In summary, this study reviews the preparation and applications of various transparent nano-ceramics in recent years, which provides a reference for low-cost and sustainable production in the future.

## 2. Transparent Nano-Ceramics for Solid-State LASERs

### 2.1. Brief Introduction

Solid-state lasers play a leading role in the field of laser application because they have the advantages of high peak power, high efficiency, long service life, safety, and reliability. In solid-state lasers, the gain medium has the most significant influence on the laser’s output performance. Doped yttrium aluminum garnet (YAG) has the advantages of high thermal conductivity, high melting point, stable chemical properties, high mechanical strength, and high creep resistance. Compared with a single crystal and glass, the main advantages of YAG transparent nano-ceramics are: (1) the process of high concentration doping is simple, which can easily improve its properties; (2) it is easy to prepare ceramics with a large size and complex shape; (3) the preparation cost is low and the cycle is short; (4) it is convenient to realize special structures and functions. Therefore, it is the focus of research in the academic community, and many countries have invested a lot of human and material resources. Nowadays, YAG transparent nano-ceramics are widely used in the gain medium of solid-state lasers. According to the literature, the performance of transparent nano-ceramics is related to the controllable doping of rare earth ions and the size of nano-powder, which are critically reviewed in this section to elaborate on the progress of transparent nano-ceramics for solid-state lasers.

### 2.2. Doped YAG Transparent Nano-Ceramics

**Figure 3:**
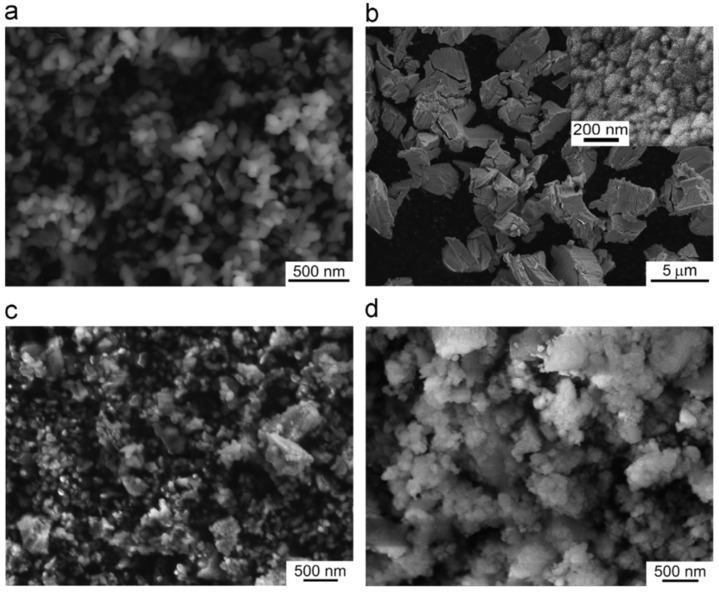
SEM images of Al_2_O_3_ (**a**), Y_2_O_3_ (**b**) starting nano powders, as well as 2.88 Y_2_O_3_–0.12Nd_2_O_3_–5Al_2_O_3_ powder mixture (**c**) and Y_2_O_3_ powders after planetary ball milling for 15 h (**d**) [16]. Reprinted with permission from Ref. [16]. Copyright 2014, copyright ELSEVIER.

**Nd^3+^-doped**. In 1995, Nd:YAG transparent ceramics for solid-state lasers with continuous wave (CW) laser emissions were first reported by Ikesue and Kinoshita [12], and the nano powers of Y_2_O_3_, Al_2_O_3_, and Nd_2_O_3_ were used as starting materials with the average particle diameters of 60, 400, and 500 nm, respectively. The optical scattering loss of Nd:YAG was about 0.9%/cm. The experiment demonstrated that the performance of solid-state lasers could be obtained with an oscillation threshold of 309 mW and a slope efficiency of 28%, respectively. In 2002, Lu et al. [13] developed Y_3_Al_5_O_12_ optical ceramic materials based on highly transparent nanocrystalline YAG. The pore volume concentration of YAG transparent ceramics was 1 ppm, and the average diameter of particles was about 10 μm. The grain boundary width was only about 1 nm. The results showed that in the preliminary comparative laser experiment of Nd:YAG ceramic and single-crystal rods, the output power of 88 and 99 W were obtained, respectively. This means that it could be used in high-power, solid-state lasers. Compared with single-crystal Nd:YAG, the light-to-light efficiency of Nd:YAG transparent ceramics with nanocrystalline needs to be further improved. However, it will become a good substitute for the widely used Nd: YAG single crystal, due to its low manufacturing cost, for different types of solid-state lasers. In 2010, Suárez et al. [14] first obtained 1 at.% Nd:YAG nano powder with an average particle size of 100 nm by using a reverse-strike precipitation method. Then, the Nd:YAG transparent nano-ceramics were prepared by the hot isostatic pressing (HIP) method. They found that the optical properties were significantly different with different sintering and HIP parameters. The infrared transmittance of the fabricated sample was 80%, and its emission spectrum was the same as a 1 at.% Nd:YAG single crystal. In 2011, Stevenson et al. [15] sintered Nd:YAG transparent ceramics at 1600 °C with B_2_O_3_ and SiO_2_ double-sintering additives. They adopted the solid-state reaction method to prepare the Nd:YAG transparent ceramics and *α*-Al_2_O_3_ (>99.99%, 100–300 nm), Y_2_O_3_ (>99.999%, 50 nm), and used Nd_2_O_3_ (>99.99%, 200 nm) nano powders as the starting materials. Additionally, the B^3+^: Si^4+^ atomic ratio ranged from 0.5 to 2 while keeping the total doping level at 1.35 mol%. The results demonstrated that the relative density of the samples exceeded 99.9% and the transmittance in the visible band was as high as 84%. They also found that densification could be completed at about 100 °C lower than the normal sintering temperature since B^3+^ greatly improved the driving force of densification. In 2014, Yavetskiy et al. [16] also utilized the solid-state reaction method to fabricate a Nd:YAG transparent ceramic, and investigated its phase formation and densification mechanism in the sintering process. As depicted in Figure 3, the particle size of Al_2_O_3_, Y_2_O_3_ starting powders, as well as 2.88 Y_2_O_3_–0.12Nd_2_O_3_–5Al_2_O_3_ powder mixture and Y_2_O_3_ powders after planetary ball milling for 15 h, ranged from 80 to 800 nm. The results showed that using Y_2_O_3_ nano powder, under bimodal particle size distribution (D_50_ ≈ 160 nm and 400 nm), could make the shrinkage effect higher than the expansion effect in the formation of the YAG phase during sintering. Additionally, the transmittance of the prepared 4 at.% Nd:YAG sample (1 mm thick) at 650 nm was 80%, which was close to that of Nd:YAG single crystal. In addition, Zhang et al. [17] studied the effect of Nd dopant and LiF additive on the microwave dielectric and optical properties of transparent YAG ceramics in the spark plasma sintering (SPS) process in 2016. The SEM images demonstrated that the size of YAG nano powder was almost between 50 and 100 nm, and the infrared transmittance of the sample was 81.8% after sintering at 1360 °C. In 2021, Jia et al. [18] comparatively analyzed the influence of tetraethoxysilane (TEOS) additives on the sintering kinetics of Nd:YAG transparent ceramics. The vacuum sintering method was used to evaluate the densification process and sintering kinetics of Nd:YAG transparent ceramic samples. The densification rate of ceramic samples rose dramatically when the amount of TEOS was raised from 0 to 3.0 wt.%. The experiment showed that the transmittance of the 0.5 wt.% TEOS sample reached 75% in the near-infrared region.

**Ho-doped**. Under direct pumping, the Ho^3+^ ion emits a quasi-three-level emission at 2.0 μm, exploited for efficient CW lasing [19,20]. Additionally, infrared lasers have also been made with Ho:YAG transparent ceramics. In 2015, Bagayev et al. [21] fabricated nano powders generated by laser ablation and then used two ways to make Ho:YAG transparent ceramics. The nano powders were made up of near-spherical particles with an average size of 8–14 nm and specific surface areas of 83.8 and 46.0 m^2^/g for the Al_2_O_3_ and Ho:Y_2_O_3_ particles, respectively. The results revealed that the transparent ceramics produced by their proposed method had better transmittance (82%) in the infrared band. Additionally, the slope efficiency of laser oscillations in the fabricated Ho:YAG transparent ceramic sample (1 mm thick) for pumping power was 40% (at 1.85 μm). In 2018, Zhao et al. [22] demonstrated a Ho:Y_2_O_3_ ceramic laser with high power, which fabricated Ho:Y_2_O_3_ ceramics by vacuum sintering and HIP methods. The in-band pumping method produced a 2117 nm laser with an output power of 24.6 W, nearly an order of magnitude higher than other ceramics. For high-power, solid-state lasers, therefore, Ho-doped sesquioxide ceramics are ideal materials.

**Er-doped**. Er-doped YAG transparent ceramics have very low levels of quantum defects, and their laser behavior is IR transitions at 1.5 and 3 μm. In 2011, Zhang et al. [23] demonstrated a 0.5 at.% Er:YAG ceramic laser, which exhibited CW emission at 1617 nm and had a slope efficiency of 51.7%. In 2015, Zhang et al. [24] reported a passively Q-switched ceramic Er:YAG laser using a saturable absorber, which emitted 1617 nm. The experimental result confirmed that the laser could reach a peak power of 11.3 kW. In 2018, a laser adopted by 0.5 at.% Er:YAG transparent ceramics, with a resonantly pumped eye-safe, was developed by Bigotta et al. The fabricated ceramics adopted a two-step approach, combining SPS+HIP methods [25]. In their study, high-purity 0.5 at.% Er^3+^:YAG powder with a specific surface area of 7 m^2^/g and an average size of 271 nm was used. The experimental results confirmed that the light-light efficiency of this laser was 20%, and the maximum slope efficiency was 31%.

**Tm-doped**. The Tm^3+^ concentration should be at least 6% to guarantee efficient down-conversion energy transfer [26]. Experiments showed that the transparent ceramics doped with Tm^3+^ have good light transmittance [27]. Zhang et al. [28] prepared highly transparent Tm:YAG ceramic by solid-phase reaction and vacuum sintering and studied its optical properties, microstructure and laser properties. Zou et al. [29] developed a high-efficiency, continuous-wave Tm:YAG transparent nano-ceramic laser pumped using a Ti:sapphire laser. Output power of up to 860 mW was produced with an absorbed pump power of 2.21 W at 785 nm, equating to a slope efficiency of 42.1% and a light-to-light efficiency of 22%. Zhan et al. presented a 2.7 mm long passively mode-locked laser based on 6 at.% Tm:YAG ceramics [30]. The pulse duration was 55 ps, and the highest output power was 116.5 mW at 2007 nm. Based on these findings, Tm:YAG transparent nano-ceramics looked to be promising candidates for ultrafast lasers with high power densities and high-efficiency output.

**Yb-doped**. The Yb^3+^-doped ceramics’ spectral properties ensure nearly pure four-level lasing, which can be easily controlled by adjusting the ambient temperature or the temperature inside the pumped lasing medium. In 2008, Nakamura et al. [31] developed a CW laser based on Yb:YAG transparent ceramics. With a slope efficiency of 72%, a 6.8 W CW output power was obtained, and the transverse intensity distribution of the Yb:YAG ceramic laser beam was a Gaussian beam. In 2012, Luo et al. [32] used Yb:YAG ceramics and a 940 nm fiber-coupled laser diode to accomplish CW lasing at 1030 nm. The basic materials were commercial Al_2_O_3_ powder (99.99 percent purity, 250 nm) and co-precipitated Y_2_O_3_ and Yb_2_O_3_ powders (60–80 nm, 9.5–10.0 m^2^/g). For a 3 mm-thick mirror-polished Yb:YAG ceramics sample, in-line transmittances at 1300 nm and 400 nm were measured to be 83.6 and 81.8%, respectively. The slope effectiveness of this laser was 62.7% according to the testing data.

Table 1 summarizes the doped YAG transparent nano-ceramics, which are described in the text grouped by doped type and publication year. It can be drawn that doped YAG laser transparent nano-ceramics have a short preparation period, low production cost, large-scale production, and high doping concentration.

### 2.3. Application

**Figure 4:**
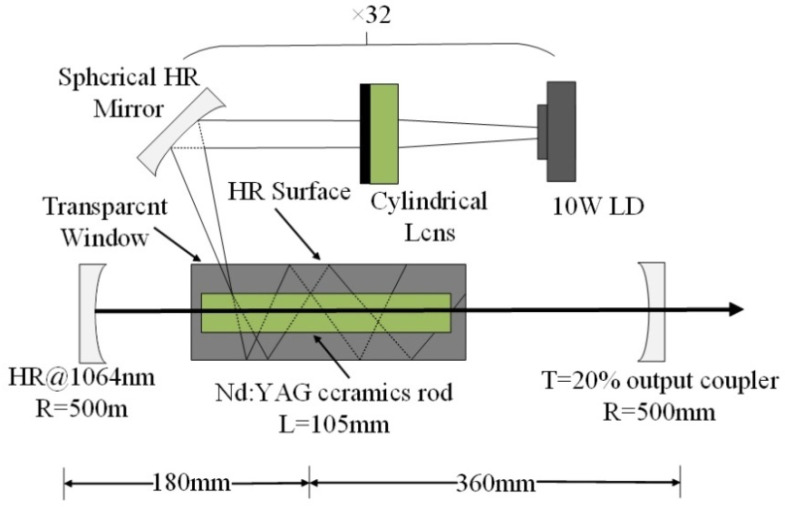
Schematic diagram of YAG transparent nano-ceramics’ application in laser diode pumping system.

The schematic diagram of YAG transparent nano-ceramics’ application in a laser diode pumping system is shown in Figure 4. As depicted in Figure 4, a symmetrical ring pump source was created using 32 groups of laser diodes (the highest output of an LD at 807 nm was 10 W) and a ϕ4 mm × 105 mm 0.6% Nd:YAG transparent nano-ceramic rod. Then, a high-power Nd:YAG ceramic laser with CW 1.46 kW was developed [13], and this was the first time that the output power of a ceramic laser exceeded the kilowatt level. The experimental findings showed that increasing the pump power to 290 W resulted in an 88 W multimode CW laser output. This meant that the light-to-light efficiency of YAG transparent nano ceramics was about 30%. In 2010, Marsh Corporation in the United States used multiple Nd:YAG transparent ceramic slabs with composite structures to achieve a laser output of more than 100 kW using direct pumping technology, of which the output power of a single Nd:YAG slab could reach 17 kW [33,34]. Nakamura [31] developed a high-power efficient transparent ceramic Yb:YAG laser with a Yb concentration of 9.8%, a pumping power of 13.8 W, a T = 10% output coupler, and a cavity length of 20 mm at a room temperature of 20 °C. At a maximum output power of 1.6 W, the ceramic Yb:YAG laser showed continuous tunability in the spectral region of 63.5 nm from 1020.1 to 1083.6 nm. A high-power passive Q-switched Ho:YAG ceramic laser was created by Yuan et al. [35]. The maximum pulse energy of this laser was 0.94 mJ, the pulse width was 28 ns, and the peak power was 33.5 kW at a pulse-repetition frequency of 28.8 kHz.

### 2.4. Summary

Since it took Ikesue and Kinoshita [12] 31 years to use lasers for Nd:YAG transparent nano-ceramics in 1995, this was not a rapid development. Over the next seven years, advances in powder synthesis and ceramic sintering allowed the 1 kW output power threshold to be broken in 2002 [13], followed by another seven years until the 100 kW mark was crossed in 2009 [33,34].

In terms of powder-preparation methods, the most mature technologies are the solid-state reaction method and liquid-phase coprecipitation process. The solid-state reaction method has a simple process, but the commercial raw material powder used has low sintering activity, which is not conducive to the densification of transparent ceramics. In terms of doped YAG transparent ceramics’ sintering, vacuum sintering is currently the most commonly used sintering technology for fabricating them. Although vacuum sintering helps to eliminate pores and improve the density, it is nevertheless unable to entirely eradicate residual pores inside the ceramics, resulting in most sintered samples having a transmittance of less than 80%. In addition, according to the needs of solid-state lasers, rare earth ion-doped YAG transparent ceramics can be used to make laser materials with excellent performance, which are widely used in the field of solid-state lasers.

It can be seen from the above experiments that after doping with Nd^3+^, the transmittance increases to 81.8%; the output power of Ho-doped sesquioxide ceramics is nearly an order of magnitude higher than that of other ceramics; Er-doped YAG transparent ceramics have very low levels of quantum defects, and their peak power can also reach 11.3 kW; Tm-doped ceramics can ensure an efficient step-down version of energy transfer; and Yb doping can adjust the environment and the temperature inside the pumping laser medium. In general, the transmittance and output power of ceramics doped with YAG were greatly improved, and the temperature could be controlled at the same time, which makes them a good candidate for ultrafast lasers with a high power density and high power output.

However, most of the current research on Nd:YAG transparent ceramics is based on experimental results [36,37], and there is a lack of relevant theoretical simulation data. For example, there is still a lack of research on the relationship between structural defects (such as grain boundaries) and the photothermal damage of ceramics, as well as on the types and concentration distributions of doped rare earth ions. In addition, it is also necessary to study the occupancy mechanism and distribution of dopant ions of different types and concentrations inside the ceramic, as well as the influence of the surrounding crystal field. These breakthroughs in the mechanism of action need to be solved through effective theoretical models.

## 3. Magneto-Optical Transparent Nano-Ceramics

### 3.1. Brief Introduction

Magneto-optical material is a new type of optical functional material that has a magneto-optical effect in the ultraviolet to infrared band. Optical devices, such as magneto-optical switches, magnetometers, and magneto-optical sensors, with various functions, are made by using the magneto-optical properties of these materials and the interaction and conversion of light, electricity, and magnetism. According to the type of materials, they can be divided into magneto-optical glass, magneto-optical crystal, magneto-optical transparent ceramics, etc. The thermal conductivity of magneto-optical ceramics is equivalent to that of magneto-optical crystals, and the thermal diffusion performance is good, which can effectively prevent thermal damage in the process of using lasers. Compared with crystals, magneto-optical ceramic materials can more easily obtain larger sizes and can be made into large-diameter magneto-optical components, with high fracture toughness and good thermal shock resistance [37]. This means that magneto-optical transparent ceramics is a new type of magneto-optical dielectric material in recent years with the advantages of a large size, Verdet constant, high laser damage threshold, and high thermal conductivity [38,39,40]. At present, the reported magneto-optical transparent ceramic materials mainly include TGG transparent nano-ceramics and TAG transparent nano- ceramics.

### 3.2. TGG

**Figure 5:**
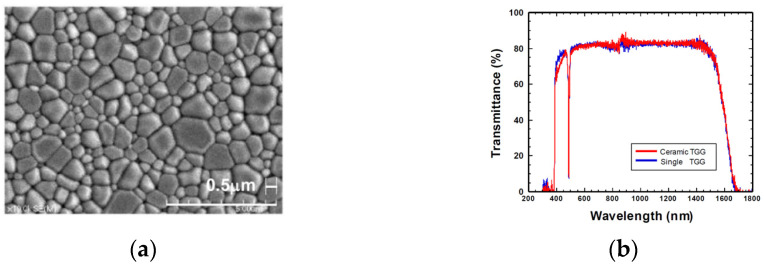
Microstructure of TGG transparent nano-ceramic and its transmittance spectra [39]; (**a**) SEM image showing the microstructure of a typical TGG transparent nano-ceramics sample; (**b**) Comparison of transmission spectra between TGG nano-ceramics samples and TGG single crystal. Reprinted with permission from Ref. [39]. Copyright 2011, copyright IEEE Xplore.

In 2003, Khazanov [41] reported TGG transparent nano-ceramics for the first time. The results demonstrated that the emergence of high-quality nano-ceramics and the improved Faraday device made it possible to apply TGG transparent nano-ceramics in higher-power lasers. In 2007, Yasuhara et al. [42] reported the Faraday effect of TGG ceramics for the first time. They tested the Verdet constant of TGG transparent nano-ceramics samples at 1053 nm as a function of temperature and found that the Verdet constant was 36.4 rad/(T·m) at room temperature, which was 87 times greater than that at the temperature of liquid nitrogen (77 K). This showed that under the same magnetic field, the length of the Faraday material could be shortened to 1/87, which provided advantages for femtosecond short-pulse lasers. In addition, the test data also confirmed that the Verdet constant of TGG transparent nano-ceramics was similar to that of TGG single crystals. In 2011, Yoshida et al. [39] systematically studied the optical characteristics and Faraday effect of TGG ceramics at room temperature. As shown in Figure 5a, the grain size distribution of the prepared TGG transparent nano-ceramics was relatively uniform, and the grain size was between 300 nm and 3 μm, which was smaller than that of the YAG transparent nano-ceramics. At the same time, they tested the optical transmittance of TGG ceramic samples in the visible and near-infrared bands and compared them with TGG single crystals. It could be drawn from Figure 5b that in the 600–1400 nm band, the transmittances of TGG ceramics and single crystals were almost equal, with both higher than 80%.

**Figure 6:**
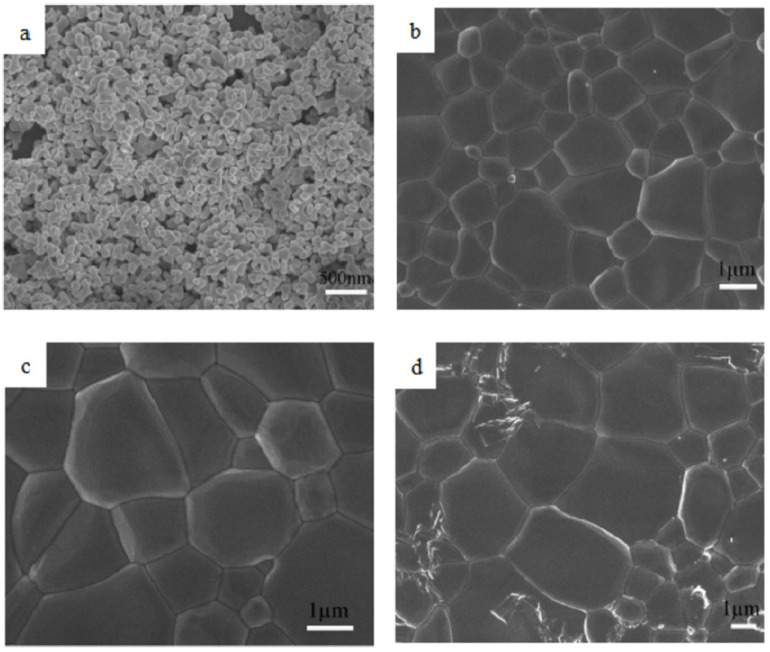
SEM morphology of TGG powders sintered at 1100 °C (**a**), the surface of sample #1 sintered at 1500 (**b**), sample #2 sintered at 1550 °C (**c**), and sample #3 sintered at 1600 °C (**d**) [43]. Reprinted with permission from Ref. [43]. Copyright 2019, copyright ELSEVIER.

In 2019, Jin et al. [43] prepared the magneto-optical properties of TGG transparent nano-ceramics. The microstructures of the TGG powders showed that the average particle size was about 80 nm, and the powder agglomerated through small connections to the particle necks, which facilitated the densification of the ceramic body. The best microstructure of the nano-ceramics with average grain sizes of 5.32 μm, fabricated by co-precipitated method and vacuum sintering methods, could be obtained while the sample was sintered at 1550 °C (in Figure 6). Additionally, they found that the best optical transmittance of TGG ceramics was close to 80% in the region of 400–1500 nm, and that the Verdet constant of the fabricated TGG ceramics decreased linearly with an increase in temperature. The results demonstrated that TGG transparent nano-ceramics could meet the requirements of magneto-optical devices working the visible-near-infrared region. In 2021, Li et al. [44] investigated the fabrication and evaluated the performance of novel TGG transparent nano-ceramics, which were doped by rare earth (RE) of Pr, Tm, and Dy. The microstructure of the powder particles was several hundred nanometers (300~700 nm) in length and several nanometers in width. After two-step sintering, no second phase was detected in the microstructure, although residual pores in the ceramic could be noticed. Therefore, the prepared ceramics all had good optical quality, and the online transmittance at 1070 nm was greater than 80%. The Verdet constant of RE:TGG transparent nano-ceramic samples (−143 rad/T·m at 632.8 nm) was optimized by rare earth doping, which was about 5% higher than that of TGG transparent nano-ceramics.

**Figure 7:**
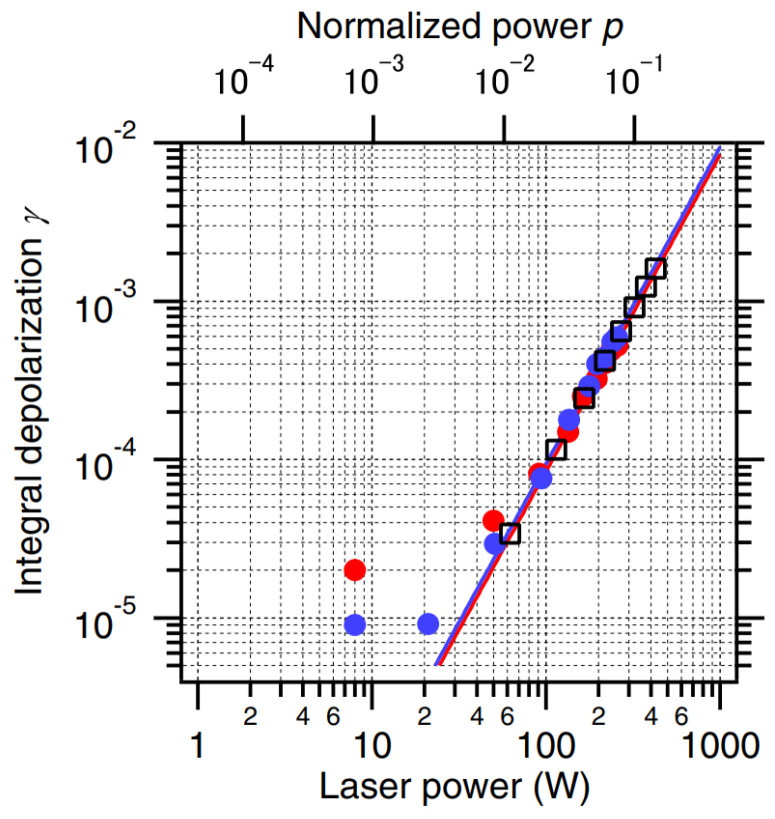
Experimental results of depolarization as a function of laser power (red and blue circles represent results for TGG ceramics with and without magnetic field, respectively; squares represent calculated results for TGG single crystals; solid lines represent theoretical curves) [48]. Reprinted with permission from Ref. [48]. Copyright 2014, copyright Optical Society of America.

With the increase in power of the CW laser and pump laser, the thermal effect of optical elements in a laser system becomes more and more serious [45,46,47]. Thermally induced birefringence reduces the isolation ratio of the Faraday isolator, which limits its use in high-power lasers [39]. In 2014, Yasuhara et al. [48] developed a TGG Faraday rotator for a high-power (257 W) laser with an isolation ratio of 33 dB and studied its thermal depolarization effect and thermal lens effect under laser irradiation. They tested the thermally induced depolarization ratio of TGG transparent nano-ceramics under laser irradiation, as shown in Figure 7. The results showed that the depolarization ratio of TGG ceramics at 257 W laser power was 5.48 × 10^−4^ under the applied magnetic field (45° Faraday rotation), and the corresponding isolation ratio was 33 dB. This confirmed that the thermally induced depolarization of TGG ceramics was almost the same as that of a single crystal. The results showed that the Faraday isolator based on TGG transparent nano-ceramics had basically met the requirements of service under a high-power laser.

### 3.3. TAG

**Figure 8:**
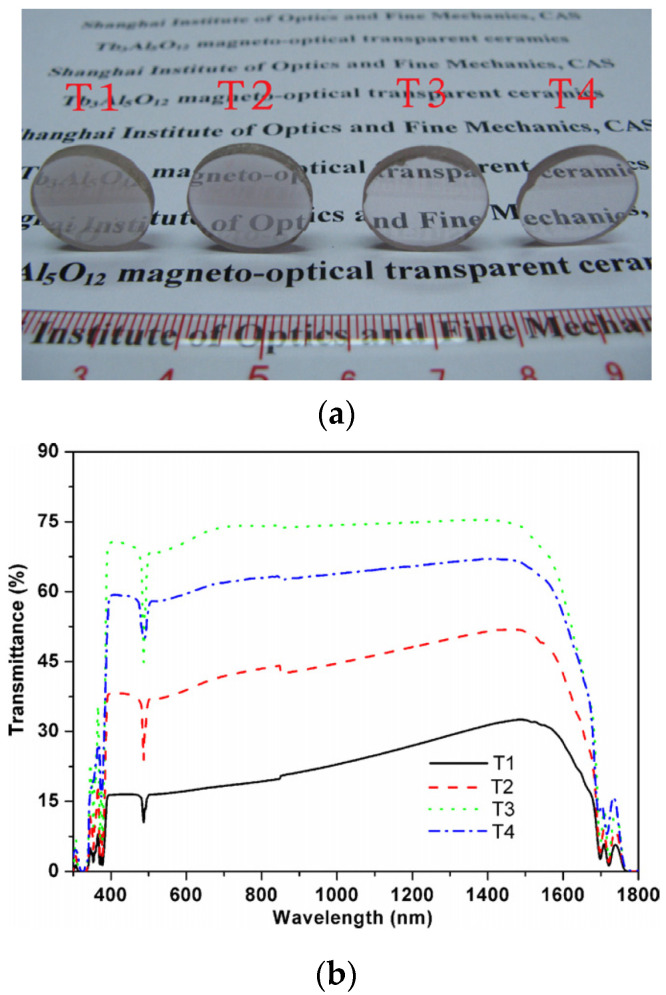
Optical quality of TAG transparent nano-ceramics; (**a**) samples and (**b**) optical transmittance of polished TAG transparent nano-ceramics [49]. Reprinted with permission from Ref. [49]. Copyright 2011, copyright ELSEVIER.

TAG transparent nano-ceramics have the same garnet structure and similar optical and thermal properties as TGG transparent nano-ceramics, but their Verdet constant is about 30~50% higher than that of TGG transparent nano-ceramics. Therefore, TAG transparent nano-ceramics have better magneto-optical properties and can be used in Faraday magneto-optical materials in visible and near-infrared bands. In 2011, Lin et al. [49] prepared TAG transparent nanoceramics for the first time by solid-phase reaction and vacuum sintering (depicted in Figure 8a) and studied the optical quality and microstructure of the samples. The samples sintered at 1650 °C had relatively good optical transparency between 400 nm and 1600 nm (up to 70%, as shown in Figure 8b). The experimental data demonstrated that the thermal conductivity of the prepared TAG transparent nano-ceramics was 6.5 W/m∙K (at room temperature), and the Verdet constant could reach −172.72 rad/T∙m (at 632.8 nm) with the best quality, which was 28.9% larger than that (−134 rad/T∙m) of the TGG single crystal [50]. This indicates that TAG transparent nano-ceramics have better magneto-optical properties than TGG single crystals and have potential commercial values. In 2015, Chen et al. [51] found that by optimizing the sintering aid, TEOS combined with MgO as a sintering aid could improve the optical quality of TAG transparent ceramics but had no effect on the magneto-optical properties. The study found that when the addition of TEOS was 0.4 wt.% and the addition of MgO was 0.1 wt.%, the optical transmittance of the obtained TAG ceramics exceeded 80% in the 500–1500 nm band, and the optical quality was greatly improved. Moreover, in 2017, Duan et al. [52] adopted the reaction sintering method combining muffle furnace pre-sintering and HIP sintering and obtained TAG transparent ceramics with ideal optical quality for the first time without vacuum sintering. After ball milling, the raw material powders were uniformly mixed, wherein the particle size of Al_2_O_3_ was 260 nm, and the particle size of Tb_4_O_7_ was 1.15 μm. The experimental results confirmed that the optical transmittance of the prepared 0.4 wt.% TEOS:TAG transparent nano-ceramics in the visible and near-infrared regions could reach more than 80%, which was very close to the theoretical limit.

In addition, some researchers found that the Verdet constant of TAG transparent nano-ceramics could be changed by adding rare earth elements [38,53,54]. In 2012, Chen et al. [53] adopted solid-state reaction and vacuum sintering methods to prepare Y^3+^ and Ce^3+^ rare earth element-doped TAG transparent nano-ceramics. They found that the Verdet constant of Y^3+^-doped TAG transparent nano-ceramics was −108.79 rad/T·m at 632.8 nm, which was smaller than that of TAG transparent ceramics. This indicated that diamagnetic ion (Y^3+^) doping would have an adverse effect on the magneto-optical properties of TAG transparent ceramics. However, the magneto-optical properties of the Ce^3+^-doped TAG transparent nano-ceramics were greatly improved, and its Verdet constant, measured as −199.55 rad/T·m at 632.8 nm, was about 16% higher than that of TAG transparent ceramics. A year later, Chen et al. [54] continued to study the relationship between the sintering process parameters and optical properties of Y^3+^-doped TAG transparent nano-ceramics, and they found that the samples sintered at 1680 °C showed the best optical properties, obtaining transmittance of 75% in the range of 900 to 1600 nm. Additionally, X-ray diffraction (XRD) results showed that samples had pure garnet crystal structure without secondary phases.

### 3.4. Others

Some sesquioxides also have magneto-optical effects and generally have high thermal conductivity, which is also a potential application value, such as in high-energy lasers [38,55,56,57]. For example, at 1064 nm wavelength, the optical transmittances of fully doped and 10% doped Y^3+^:Y_2_O_3_ ceramics were 63.4 and 79.3%, respectively, but both were lower than their theoretical transmittances of 81.1% [55]. The Ho_2_O_3_ magneto-optical transparent nano-ceramic was successfully prepared by the SPS sintering method, and its transmittance at 1 μm was measured to be about 60%, but its Verdet constant at the wavelength of 1064 nm was −46.3 rad/(T·m), which was close to that of TAG, which was about 1.3 times that of TGG [56]. These studies [55,56,57] showed that yttrium oxide (Y^3+^:Y_2_O_3_), holmium oxide (Ho_2_O_3_) [58], dysprosium oxide (Dy_2_O_3_), and other nano-transparent ceramics still need to be further optimized to improve their optical properties.

### 3.5. Potential Application

Transparent nano-ceramic is the core of modern magneto-optical materials, which provides a broad development prospect for the Faraday isolator. Furuse et al. [59] compared characteristics of Faraday isolators with different materials of magneto-optical medium, which are listed in Table 2. It can be seen from Table 2 that there is still a certain gap in the isolation between TGG nano-ceramic ceramics and TGG crystals. TGG crystals can obviously work effectively in higher-power lasers and achieve a stable isolation ratio. Although the isolation measurement power is lower for TAG and Ce:TAG transparent nano-ceramics, it can be estimated by calculation that the extinction ratio of the TAG ceramic samples can be maintained above 30 dB when the laser power reaches the kilowatt level.

**Figure 9:**
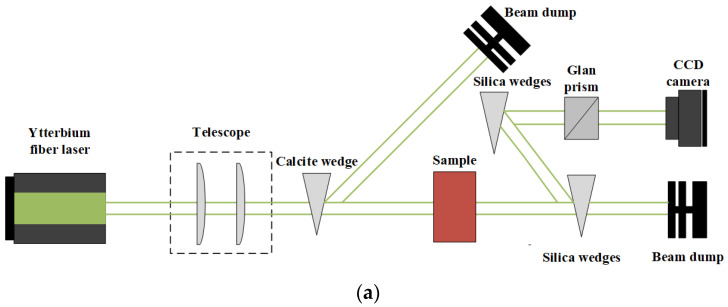

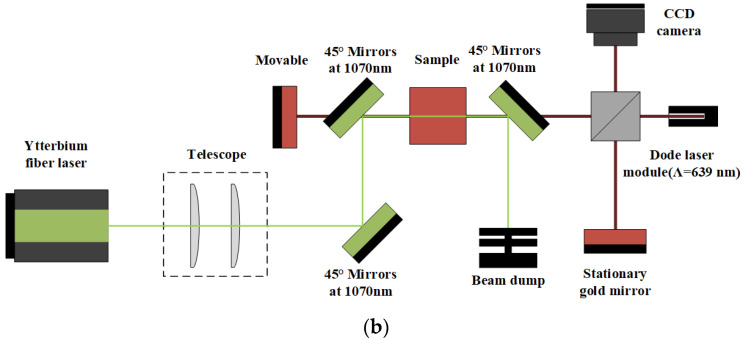
Scheme of the experiment of (**a**) thermal depolarization measurement and (**b**) studying thermally induced lens.

In 2021, Starobor et al. [60] fabricated a TGG/sapphire composite magneto-optical element Faraday isolator, whose maximum operating power with an isolation ratio higher than 30 dB was estimated to exceed 2 kW, which was almost three times that of a single TGG crystal. In addition, they also performed experimental (shown in Figure 9) and numerical studies on thermally induced depolarization and thermal lensing of composite elements, and the optimized structure could be successfully operated at a high radiation power. Experimental results showed that an isolation ratio of 34 dB in the composite elements was achieved at a laser power of 700 W, which was 5 dB higher than the classical single element. This indicated that TAG transparent nano-ceramics could be used to prepare kW Faraday isolators.

### 3.6. Summary

With the continuous improvement of techniques, the optical quality of transparent nano-ceramics has been greatly improved and is almost comparable to that of its crystals [61,62]. Among the magneto-optical ceramic materials that have appeared so far, the research focus is mainly on TGG and TAG transparent nano-ceramics. As can be seen from the research results of the existing literature, most of the TGG and TAG transparent nano-ceramics have a light transmittance of 80% in their expected working band, and the Verdet constant meets the working requirements, which potentially gives them commercial value in large-scale applications. In addition, the magneto-optical properties of TAG transparent nano ceramics are changed by doping different rare earth elements. This can not only increase the Verdet constant but also could reduce the Verdet constant, which can regulate its magneto-optical characteristics by controlling the type and proportion of rare earth elements. This research also needs attention in the future study of TGG transparent nano-ceramics.

## 4. Transparent Armor Nano-Ceramics

### 4.1. Brief Introduction

**Figure 10:**
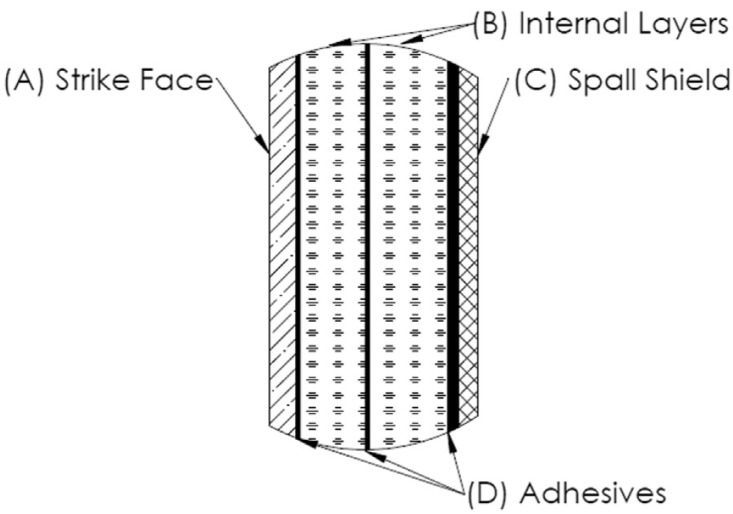
Scheme of functional layers in a transparent armor concept with a four-layer design [65]. Reprinted with permission from Ref. [65]. Copyright 2017, copyright ELSEVIER.

Transparent armor, such as face shields, windows of military vehicles, and lookout windows of aircraft, is one of the most important personnel protection technologies. Currently available transparent armor consists of several layers and is very thick to resist the ballistic impact of multiple hits. Today, the demands placed on these systems are increasing [63,64]. As a result, traditional glass-based armors have become impractical in terms of weight and thickness constraints, which has led to increased consideration of transparent ceramics for such applications. As shown in Figure 10, a versatile four-layer design (A—projectile erosion fragmentation layer; B—energy absorption, crack arrest layer, C—fragmentation protection layer; and D—adhesive layer) can be used for the development of light armor, where the first layer is the core layer in the overall four-layer design [65]. When transparent nano-ceramics such as MgAl_2_O_3_ or AlON are used for this functional layer, the weight and thickness of glass-made armor can be reduced by about 30–60% [66]. In addition, some weapon systems that need to withstand harsh environmental conditions also require relatively large windows and domes [67,68,69].

### 4.2. MgAl_2_O_4_

Due to its high hardness, strong chemical resistance, and high transparency in the UV-Visible and mid-IR range, MgAl_2_O_4_ transparent nano-ceramics are expected to be used in optical components and defense applications [64], such as optical lenses, aircraft/vehicle windows, and missile domes [70]. Moreover, they can achieve a balance between optical performance and production cost [71]. In 1974, Bratton [72] fabricated translucent polycrystalline MgAl_2_O_4_ ceramics with co-precipitated spinel as raw material, doped with 0.25 wt.% CaO as a sintering aid. The experimental results showed that at a certain temperature, the relative density of sintered spinel could reach 99.7% (close to the theoretical value of 100%) and that the transmittance in the visible light region was between 67 and 78%.

**Figure 11:**
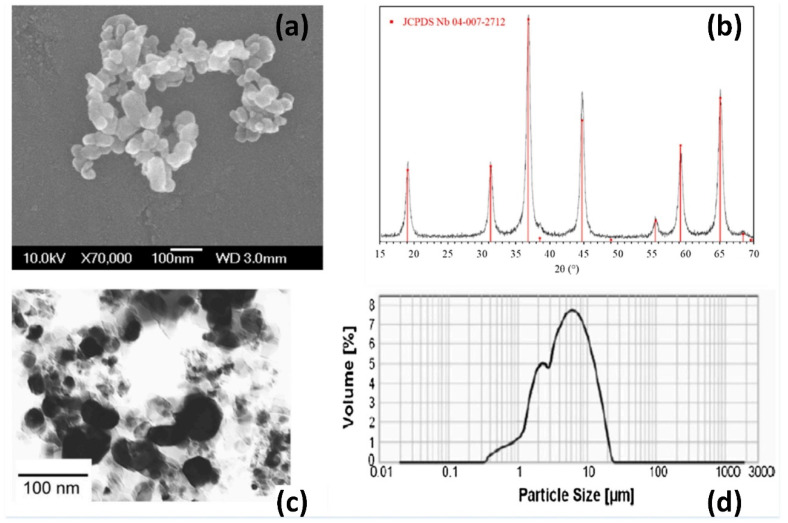
Sample particle size distribution and XRD pattern; (**a**) FEG-SEM micrograph, (**b**) XRD pattern, (**c**) TEM micrograph, and (**d**) particle size distribution of as-received spinel powder SPI-P1 [77]. Reprinted with permission from Ref. [77]. Copyright 2021, copyright ELSEVIER.

To obtain MgAl_2_O_4_ transparent nano-ceramics with high optical transparency, high-quality precursor powders (average particle size of 150 nm [73]) and optimized concentrations of sintering aids, such as CaO [72], B_2_O_3_ [73], and LiF [74], are required. In 2013, Esposito et al. [75] fabricated MgAl_2_O_4_ transparent nano-ceramics by hot pressing and investigated their characterization. They prepared two starting powder mixtures (with a purity level greater than or equal to 99.99%) made from commercial Al_2_O_3_ and MgO products, taking into account the stoichiometric ratio of MgAl_2_O_4_. Experimental results showed that the value of D_50_ of MgAl_2_O_4_ was close to 180 nm. The particle size of the starting powder had a slight effect on the sintering evolution and final microstructure but not uniformly on the final transmittance. Optical inspection experiments showed that up to 70% transmittance (the highest value of 78% in the 1100 nm band) was obtained in visible light. In addition, thermodynamic studies of the reactions of LiF, MgO, and Al_2_O_3_ could help to understand the densification mechanism that affected the transmittance of spinel. In 2015, Esposito et al. [76] investigated the effect of the pressure applied during sintering on the final optical properties of the MgAl_2_O_4_ transparent nano-ceramics in hot pressing. By establishing a thermodynamic model, the role of lithium fluoride as a sintering aid was clarified. The results of the study showed that transparency, close to the theoretical value, could only be achieved at very high pressures (200 MPa or higher) due to spinel destabilization. Boulesteixa et al. [77] prepared MgAl_2_O_4_ transparent nano-ceramics from pure aluminum and magnesium sulfate using colloidal chemistry. Figure 11a,c shows that the basic particles are between 40 and 80 nm in diameter and have an isotropic shape. The phase composition of the powder is shown in Figure 11b, while the particle size distribution of the as-received powder is shown in Figure 11d.

In general, it is challenging to fabricate MgAl_2_O_4_ transparent nano-ceramics using traditional pressureless sintering techniques [73,78]. Therefore, complex sintering strategies, such as hot pressing, HIP, and SPS, are often employed to develop these ceramics [74,75,76,79]. In 2010, Meir et al. [80] synthesized and densified MgAl_2_O_4_ transparent nano-ceramics using the SPS method. The alumina particles in the starting powders were well separated and polygonal in shape with a particle size of about 100–500 nm, while the magnesium oxide powder was strongly agglomerated, with a size of about 10–20 μm and a relatively high specific surface area. The addition of a sintering aid of 1 wt.% LiF to the mixed powder promoted the synthesis of spinel. The experimental results indicated that LiF vapor played an important role in eliminating residual carbon contamination and achieving a fully dense state. The optical transmittance of the MgAl_2_O_4_ transparent nano-ceramics prepared by the SPS method reached 78% in the 400–800 nm band. In 2020, Liu et al. [81] successfully prepared MgAl_2_O_4_ transparent nano-ceramics by air pre-sintering and HIP methods. They found that a small amount of CaO (0.1 wt.%) was very effective for the densification of ceramics. They also found that the relative density of MgAl_2_O_4_ transparent nano-ceramics changed from 86.3% to 99.4% when the pre-fired temperature was increased from 1450 °C to 1550 °C. Optical experimental results demonstrated that the prepared large transparent nano-ceramics, with an average grain size of 1500 nm, exhibited high transmittances of 86.3% and 82.5% at 1100 nm and 600 nm, respectively. In 2020, Liu et al. [82] used high-purity spinel nano powders, with a particle size of 55 nm and a specific surface area of 30 m^2^/g, to successfully prepare MgAl_2_O_4_ transparent nano-ceramics by microwave sintering and HIP methods. They found that microwave-sintered MgAl_2_O_4_ transparent nano-ceramics could achieve higher densities at lower temperatures and in a shorter time than conventional pressureless sintering. Experimental results showed that the in-line transmittances of MgAl_2_O_4_ transparent nano-ceramics 1.5 mm thick that were obtained by microwave sintering (1400 °C, 80 min) and HIP (1650 °C, 180 MPa, 3 h) were 86.4% and 80.2% at 1064 nm and 400 nm, respectively. Therefore, MgAl_2_O_4_ transparent nano-ceramics have excellent light transmission ability [83].

### 4.3. AlON

AlON transparent nano-ceramics have excellent light transmittance in the near-ultraviolet to mid-infrared band (0.2–6 μm), and the theoretical transmittance is as high as 85.2% [84]. In addition, they also have excellent properties, such as high hardness, high strength, high-temperature resistance, friction resistance, and acid and alkali corrosion resistance. In 1959, Yamaguch and Yanagida [85] first reported cubic spinel-type aluminum oxynitride (AlON), which was produced from a compound between alumina and aluminum nitride in a reducing atmosphere above 1650 °C. They also studied the physical constants of AlON, such as crystal structure, density, refractive index, dielectric constant, and magnetic susceptibility.

**Figure 12:**
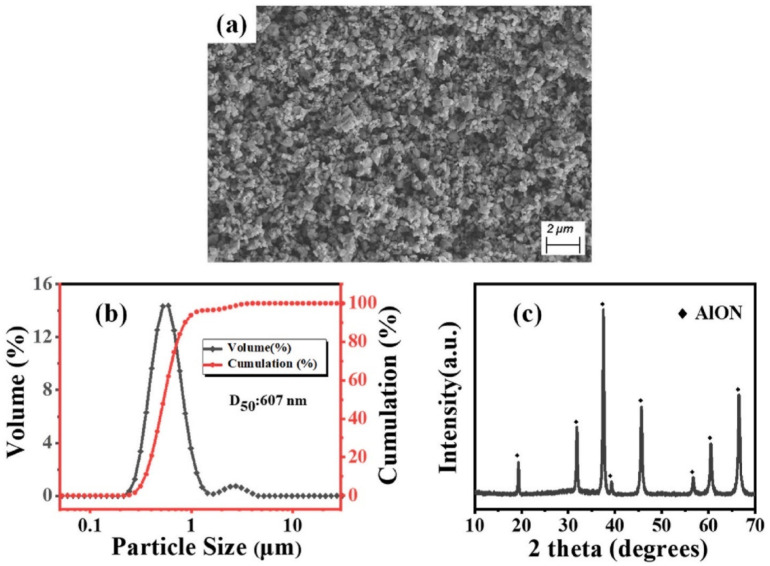
Sample size and XRD pattern; (**a**) SEM images of the morphologies, (**b**) particle size distribution, and (**c**) XRD pattern measured of the AlON powders after ball milling [89]. Reprinted with permission from Ref. [89]. Copyright 2022, copyright ScienceDirect.

First, nano powder is required to fabricate AlON transparent nano-ceramics. After obtaining the AlON nano powder, it should be fabricated by sintering at a temperature above 1850 °C for at least 20 h in nitrogen. In 2011, Qi et al. [86] adopted a two-step approach with Al_2_O_3_ and aluminum nitride (AlN) nano powders as the starting materials to prepare AlON transparent nano-ceramics. In their studies, the average particle size of nano powders of α-Al_2_O_3_, γ-Al_2_O_3_, and AlN were 80, 20, and 20 nm, respectively. Experiments showed that the AlON Nano-ceramic samples were transparent after sintering at 1880 °C for more than 5 h. With the extension of the holding time, the grain size of the sample increased slightly, while the pore size and porosity decreased obviously, so the light transmittance increased. Additionally, the sample had a transmittance of 55% (near 5 μm band) at a hold time of 20 h. While the infrared transmittance of ceramics is promising, the visible transmittance is not high enough. In 2012, Jin et al. [87] fabricated a highly AlON transparent nano-ceramic that was sintered from nano powder without pressure. During carbothermal nitridation, a layer of amorphous carbon on the surface of Al_2_O_3_ particles effectively prevented agglomeration and grain growth, and the bimodal particle size distributions of the obtained AlON powders were concentrated at 200 and 700 nm, and their maximum particle sizes were both below 900 nm. The experimental results confirmed that the AlON transparent nano-ceramics with an average online transmittance above 80% in the visible light to infrared range were obtained by a pressureless sintering method. In 2018, Zhao et al. [88] carried out a detailed investigation of planetary ball-milling for coarsened AlON nano powder. Their results revealed that the weight ratio of balls to powder, rotational speed, and planetary milling time had a significant effect on the microscopic morphology, particle size distribution, and average particle size of the powders. Using fine and uniform AlON nano powder with an average particle size of less than 300 nm and excellent sintering properties, subsequently, a sample of AlON was successfully fabricated from the finely treated powder synthesized by the carbon thermal nitriding method at 1880 °C for 6 h. The optical test demonstrated that the high performance with an online transmittance of 84% at 2000 nm of the sample could be achieved. In 2022, Zhang et al. [89] thoroughly milled the AlON powder to fully mix the nanopowder with the sintering agent. Figure 12a shows an SEM image of the particle morphology after grinding; the particle size distribution is shown in Figure 12b,c, which shows that the high-temperature decarbonization and ball-milling process do not affect the purity of the AlON powder.

**Figure 13:**
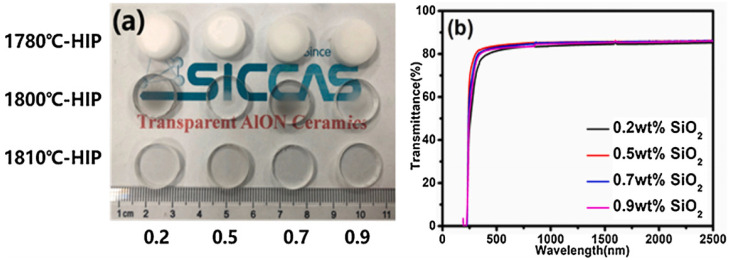
Samples and transmittance curve; (**a**) samples doped with different SiO_2_ contents and different temperatures in the process of HIP; (**b**) transmittance of AlON transparent nano-ceramics doped with different SiO_2_ contents at 1810 °C for 3 h [92]. Reprinted with permission from Ref. [92]. Copyright 2021, copyright ScienceDirect.

In the preparation of AlON transparent nano-ceramics, sintering aids are generally used to control the growth of Al_2_O_3_ grains. MgO and Y_2_O_3_ were used as co-sintering aids by Yuan et al. [90] to fabricate AlON transparent nano-ceramics by reactive sintering. The densification of AlN transparent nano-ceramics was regulated by the sintering aids and sintering duration, allowing their optical performance to be efficiently tuned. Furthermore, co-doping with MgO and Y_2_O_3_ enhanced densification more effectively than either MgO or Y_2_O_3_ alone. The optical test demonstrated that 1 mm-thick fabricated samples of AlON transparent nano-ceramics, doped with 1 wt.% MgO and 0.08 wt.% Y_2_O_3_, reached the maximum of 60% in-line transmittance at 600 nm after sintering in N_2_ for 12 h at 1950 °C. In 2018, Shan et al. [91] adopted the pressureless sintering method to fabricate AlON transparent nano-ceramics with CaCO_3_ doping. For a sample with a thickness of 2 mm, the transmittance of AlON transparent nano-ceramics doped with a mass fraction of 0.3–0.4% CaCO_3_ reached 83–85% at around 3700 nm. In the wavelength range of 200–6000 nm, the transmittance of AlON doped with CaCO_3_ was always higher than that of AlON doped with an ideal amount of Y_2_O_3_. In 2021, Li et al. [92] prepared AlON transparent nano-ceramics by HIP assisted by the dissolution of gas inclusions. They investigated the influence of additive content, pre-sintering, HIP, and annealing parameters on the performance of AlON transparent nano-ceramics. The fabricated samples and transmittance curve are depicted in Figure 13, and the sample doped with 0.5 wt.% SiO_2_ exhibited the best performance. By comparing different sintering additives and pre-sintering atmospheres, the densification mechanism of the material was studied. The prepared AlON transparent nano-ceramics maintained a high transmittance of 85.8% at 2000 nm.

As mentioned above, ALON ceramics have excellent optical and mechanical properties and extremely strong light transmittance (up to 85.2%), which makes them light-transmitting materials with excellent application prospects. Secondly, because of their good wear resistance; good scratch resistance; and light, thin, and strong properties, even if they are rubbed and damaged, the light transmittance will not be affected, so they can be used as a reinforcing material in transparent armor. In the civilian field, because of its high hardness and good durability, it can be used in the casing of precision instruments such as watches and goggles [93].

### 4.4. Potential Application

**Figure 14:**
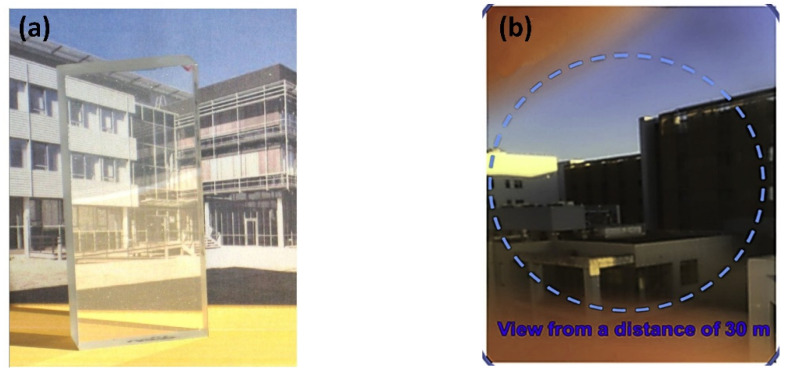
Transparent armor nano-ceramics; (**a**) MgAl_2_O_4_ transparent nano-ceramics, 12 mm thick, created through pre-sintering in air and HIP methods [83]. Reprinted with permission from Ref. [83]. Copyright 2014, copyright ScienceDirect. (**b**) a photograph revealing the transmittance of the Mg-γ-AlON transparent ceramic after HIP treatment [96]. Reprinted with permission from Ref. [96]. Copyright 2019, copyright ScienceDirect.

As depicted in Figure 14a, a large-scale MgAl_2_O_4_ transparent nano-ceramic, 12 mm thick, was fabricated by gelcasting and pre-sintering in air HIP methods, and the average grain size was 8 μm. In 1998, after realizing the mass production of powder, the research on AlON transparent ceramics progressed rapidly. At present, the largest size of AlON flat window that can be successfully fabricated is 880 × 45 × 12 mm [94]. In 2019, the Surmet company realized the production and engineering application of large-scale AlON transparent nano-ceramics in batches through pressureless sintering and HIP methods [95]. Then, a high optical quality window, fabricated by AlON transparent nano-ceramics of about 0.41 m^2^, was realized. The emergence of large-scale armored transparent ceramics makes these materials more frequently used in helicopter protection, personnel protection, infrared windows for reconnaissance, and other fields because their weight and thickness are only half of that of traditional bulletproof glass. Figure 14b shows the effect observed at a distance of 30 m from a γ-AlON transparent ceramic that was prepared by HIP post-treatment at 1800 °C for 2 h under 190 MPa [96].

**Figure 15:**
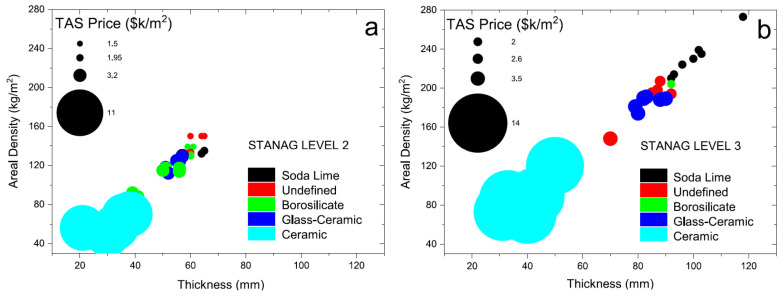
Comparison of transparent armor materials for STANAG 4569; (**a**) Level 2 and (**b**) Level 3 [65]. Reprinted with permission from Ref. [65]. Copyright 2017, copyright ELSEVIER.

Unfortunately, at present, the manufacturing cost of transparent armor nano-ceramics is relatively high. A comparison of transparent armor materials for STANAG 4569 was conducted by Benitez et al. in 2017 [65] and is depicted in Figure 15. Compared with other materials, transparent nano-ceramic materials have the lowest areal density, only half of glass–ceramic materials. However, the maximum thickness of transparent armor nano-ceramics is still difficult to achieve compared to that of other materials. In addition, the manufacturing cost is also 5 to 10 times higher than other types of materials. To promote transparent armor nano-ceramics, therefore, it is necessary to reduce the fabricating cost. This could be achieved by reducing manufacturing steps or finding other alternative materials. For manufacturing process optimization, one-step sintering methods need to be created in the future to obtain fully dense materials.

### 4.5. Summary

At present, it is difficult for MgAl_2_O_4_ transparent nano-ceramics to achieve the theoretical density required for transparency by conventional sintering methods because they are very sensitive to powder size, agglomerates, impurities, and additives. The defects of the stoichiometric ratio, impurities, particle size, and agglomerates of the starting nano powders are generally difficult to improve by adjusting the process parameters, which affect the optical properties of the prepared samples. To obtain MgAl_2_O_4_ transparent nano-ceramics with high optical transparency, high-quality starting powders are indispensable. Meanwhile, it is also necessary to optimize the concentration of sintering aids or add some rare earth elements. Existing commercial MgAl_2_O_4_ transparent nano-ceramics are sintered by pressureless sintering/HIP or hot-pressing sintering/HIP. Thereafter, the manufacturing cost of these processes is still high, and it is difficult to produce large plates.

The AlON powder synthesized in the liquid phase has relatively high purity, small particle size, uniform distribution, and relatively high chemical activity, but the synthesis process conditions are not suitable for large-scale production. The solid-phase pulverization method can ball-mill micron-sized AlON powder to the nano-scale; the preparation process is relatively simple, and it is easy to achieve large-scale production, but ball milling causes powder lattice distortion increases the defect concentration, and introduces impurities. Therefore, the sintered ceramics have special structures such as impurities, which affect the optical properties. As reviewed in Section 4.3, AlON transparent nano-ceramics usually need to be sintered for a long time above 1800 °C without external field assistance; otherwise, it is difficult to make these materials fully dense. Although preparation methods have undergone great progress in the past few decades, there are still some difficulties in the large-scale preparation process, such as shrinkage during drying and cracks forming inside the green body. In addition, the sintering of large-sized AlON transparent nano-ceramics also causes an uneven microstructure due to uneven temperatures, which in turn leads to stress birefringence and reduces their optical properties.

## 5. Other Transparent Nano-Ceramics

### 5.1. Electro-Optical Transparent Nano-Ceramics

The rise of emerging electro-optical materials is in line with the arrival of modern electronic, optical, and laser technologies, which are increasingly demanding materials. Compared with other materials, they have fast speed, low consumption, high reliability, and strong interference suppression ability, which mainly used in transducers, actuators and sensors, and it has a great impact on space structure, electronic industry, etc. [97,98]. The new electro-optic ceramic material not only has the advantages of ordinary electro-optic materials but also has a fast response speed. The response time is generally only a few nanoseconds, and the electro-optic coefficient is larger than other materials, about 2 × 10^−^^15^~6.6 × 10^−^^15^ (m/V)^2^. At present, commonly used electro-optical transparent ceramic materials are mainly two types: lead lanthanum zirconate titanate ceramics (PLZT) and lead magnesium titanate–lead titanate ceramics (PMN-PT) [99]. PLZT ceramic is a kind of transparent ceramic with an ABO_3_ type perovskite structure. It has strong light transmittance in the visible light to infrared light band. The light transmittance reaches its peak at about 600 nm, 802 nm, and 88 = 0 nm. PLZT ceramics are more commonly used in the manufacture of multi-functional, low-loss optical devices, as well as in holographic storage technology and optical fiber sensing [100]. The PMN-PT transparent ceramic is an ABX_3_ type ceramic with a high dielectric constant, which can reach up to 600 at room temperature. Additionally, it has better optical transparency. At the visible light wavelength of 400~2000 nm, the transmittance increases from 0 to 70% and finally stabilizes at 70% when the temperature rises from 400 °C to 2000 °C. It has a wide range of applications in satellite communications, sensors, frequency converters, etc. [101].

**Figure 16:**
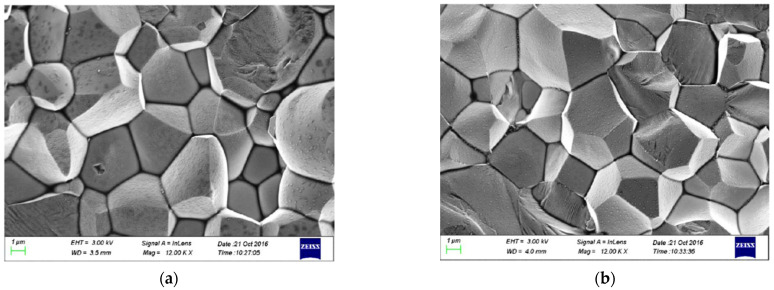

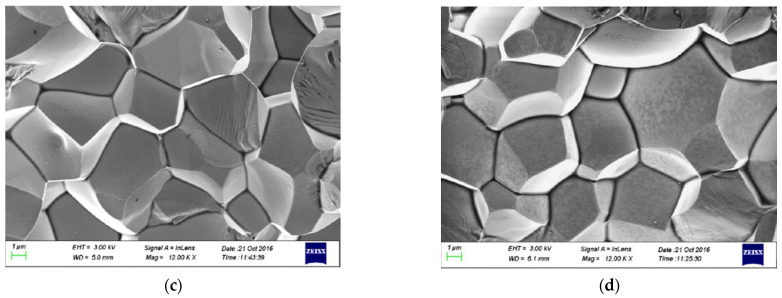
SEM images of PMN-PT polycrystals; (**a**) 1275 °C, 30 min: average grain size (2.7 µm); (**b**) 1275 °C, 60 min: average grain size (3.2 µm); (**c**) 1300 °C, 90 min: average grain size (3.8 µm); (**d**) 1300 °C, 120 min: average grain size (4.3 µm) [98]. Reprinted with permission from Ref. [98]. Copyright 2019, copyright ScienceDirect.

In 1970, Haertling et al. [102] first used the hot-pressing sintering method to prepare opto-ceramics with a thickness of 1 mm. This method could improve the compactness of ceramic materials. The density could reach about 99% of the standard density, and the light transmittance could reach 80%. In 2016, Somwan et al. [103] added powders of Bi_2_O_3_ and CuO oxides in the process of preparing PLZT ceramics and fired particles with a diameter of 1 cm without agglomeration. It was found that the dielectric constant reached a peak value of 2000 when the temperature increased to 215 °C, and then the dielectric constant began to decrease with the increase in temperature. After adding the mixture, the sintering temperature was reduced by up to 50 °C. In 2018, Samanta et al. [104] doped Nb and Fe elements during the preparation of PLZT ceramic materials, in which the radius of Fe^3+^ was 69 pm, and the radius of Nb^5+^ was 78 pm. The results showed that the conductivity increased from 10^−8^ to 10^−6^ S/cm when the frequency was increased from 100 Hz to 1 MHz at room temperature. When the doping was 2%, the energy storage density reached a maximum of 140 mJ/cm^3^. In 2017, Zhang et al. [105] prepared PMN-PT ceramics with a thickness of 0.35 mm using a two-step hot-pressing method, in which the contents of lanthanum, PMN, and PT were 3%, 75%, and 25%, respectively. When the measured temperature continued to rise, the half-wave voltage gradually increased from 200 V to 400 V, and the electro-optic coefficient continued to increase. In 2018, Wang et al. [106] reported on the preparation of PMN-PT/CFO thin films by sol–gel spin-coating technology. When the ratio of CFO to PMN-PT was 4:1, the leakage amount reached its maximum. The film quality was improved, and when the temperature increased from 650 °C to 730 °C, the leakage current decreased from 97.54 to 40.59. This indicates that the ferroelectric performance improves. The coercive electric field and the polarization curve are similar, indicating that there is some interaction between the ferroelectric phase and the magnetoelectric phase. In 2021, Ze et al. [97] prepared PMN-PT materials using a two-step sintering method. The thickness of the small ceramic was 0.85 mm, and the diameter was 10 mm. The thickness of the large ceramic was 1 mm, and the diameter was also 10 mm. After testing, it was found that as the wavelength gradually increased to 900 nm, the transmittance gradually increased to 70%. When Sm was doped, the peak value of optical power would move back. However, the basic rule was that with the increase in electric field intensity, the optical power gradually increased to 100% and then continued to decrease. Pramanika et al. [98] prepared PMN-platinum piezoelectric ceramics using the solid-state reaction method and analyzed the microstructure of the four fractured and sintered samples by scanning electron microscopy and found that their average particle sizes were 2.7, 3.2, 3.8, and 4.3 μm, respectively (Figure 16). Table 3 summarizes the representative papers on the preparation of electro-optically transparent nano-ceramics, mainly including the powder, fabrication and remarks.

At this stage, electro-optic transparent ceramics are mainly used in electro-optic modulators, high-speed electro-optic switches, and ultrasonic transducers. Next, we will analyze these devices in detail. Firstly, using the electronically controlled refraction effect in the electro-optical material, the electro-optical transparent ceramic is prepared as an electro-optical modulator, in which the basic parameters such as the frequency and phase of the light beam can be determined by the characteristics of the electro-optical material. This can provide higher working accuracy, greater working reliability, and stronger anti-interference ability. The use of this modulator can minimize the degradation of the optical fiber system and increase the life of the equipment [107]. In addition, electro-optic transparent ceramics can also be used in high-speed electro-optic switches. The main working principle is to use the electronically controlled refractive index of electro-optic materials to adjust and use optical signals to control the switch. Moreover, the electro-optic material has good light transmittance, a high electro-optic coefficient, and a low cost, which have a great guiding effect on the development of electrical components in the future. However, the LiNbO_3_ crystal is mainly used in the existing electro-optic switch. There are still some defects, and the process of selecting the crystal axis when transmitting light is more complicated [108]. Moreover, PLZT ceramic materials have small diameters, good dielectric and piezoelectric properties, and good electromechanical coupling coefficients, which can be used in medical imaging technology and ultrasonic transducers. After using the transparent optical fiber as the main component, the size of the material and the damage to the surrounding materials, such as laser cutting, can be reduced to a limited extent. Additionally, the use of this material can improve the resolution of the device and increase the working accuracy [109]. Furthermore, this study found that when PMN-PT and polyvinylidene fluoride (PVDF) materials were mixed, the piezoelectric coefficient was very high. The device’s perovskite structure with good crystallinity results in better self-powering performance, harvesting energy from the environment and reducing the cost required for the device. When subjected to external force, both PMN-PT and PVDF materials generate an electric potential to maintain the stability of the material and reduce the possibility of fracture [110].

### 5.2. Scintillation Transparent Nano-Ceramics

A scintillator is a device that can convert high-energy (X, γ) radiation or charged particles into new materials that emit visible light. According to the shape, composition, and structure of the scintillator, it can be divided into scintillating glass, scintillating ceramics, scintillating gas, scintillating crystal, scintillating plastic, etc. Among them, single-crystal and ceramic scintillators are the most widely used materials, mainly used in high-energy physics (precision electromagnetic energy), medicine (medical imaging), industrial applications (CT flaw detection), and stone well detection [111,112]. With the wide application of scintillating ceramics, scholars have carried out a series of research on its preparation method, light transmission, and scintillation performance.

**Figure 17:**
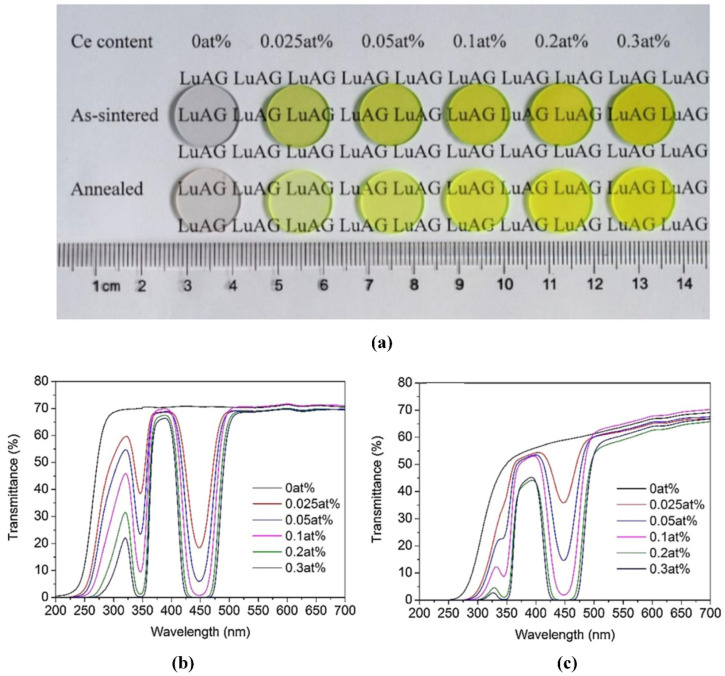
The properties of Mg:LuAG scintillation ceramics with different Ce contents (0.025~0.3 at.%) after sintering. (**a**) Transmittance of samples with different Ce contents; (**b**) Sintered state of different Ce content transmittances of Mg:LuAG ceramics; (**c**) Transmittance of annealed Mg:LuAG ceramics with different Ce contents [117]. Reprinted with permission from Ref. [117]. Copyright 2018, copyright ScienceDirect.

In 1895, a German physicist, Lunchen, stumbled upon a flashing light near a tube coated with barium platinum cyanide while conducting a cathode-ray experiment and named it an X-ray [113]. In 1896, CaWO_4_ was first used as an X-ray fluorescent powder for human body X-ray photography [114]. In 2017, Zhou et al. [115] synthesized Nd^3+^ activated SrF_2_ nanoparticles via the precipitation method and characterized the microstructure and morphology behavior of Nd^3+^-doped nanoparticles using X-ray diffraction (XRD), field emission scanning electron microscopy (FESEM), and an energy-dispersive X-ray spectrometer (EDS). They found that the synthesized powders had good sinterability, and transparent ceramics with a transmittance of about 80% at 1060 nm could be prepared by vacuum hot-pressing sintering at 800 °C for 2 h. In 2018, Yi et al. [116] prepared (Ca_0.94_Gd_0.06_) F_2.06_ transparent ceramics with Nd^3+^ content of 0.5–5.0 at.% using the vacuum hot-pressing sintering method and studied its structure, spectrum, and thermal properties. It was found that the characteristic absorption peak intensity of the Nd^3+^ ion increases linearly with the increase in its content. With an increase in Nd^3+^ content from 0.5 at.% to 5.0 at.%, the measured life dropped dramatically from 484.9 μs to 47.8 μs. In addition, they also found that the thermal properties of the ceramics were closely related to temperature and Nd^3+^ concentration. Chen et al. [117] prepared Ce, Mg:LuAG transparent ceramics (Ce concentration of 0.025–0.3 at.% and Mg concentration of 0.2 at.%) using the solid-phase method and studied the effect of Ce content on its light transmittance. As shown in Figure 17, Figure 17a represents the transmittance of six samples of varying content. They discovered that with a Ce content of 0.1 at.%, the transmittance of six Ce^3+^ samples sintered at 510 nm was close to 71%. Mg:LuAG ceramics had the best properties, as shown in Figure 17b. In Figure 17c, it can be observed that the absorption bands of Ce^3+^ increase gradually at 345 nm and 445 nm after annealing. In 2019, Hostasa et al. [118] investigated the effects of three sintering aids (MgO, CaO, and TEOS) on the densification and optical properties of Ce:GGAG transparent ceramics. The experimental results showed that the three additives tested increased the density of Ce:GGAG after sintering, but only TEOS provided sufficient densification to result in transparency. In 2020, Trofimova et al. [119] studied the radioluminescence (RL) properties of Lu_3_Al_5_O_12_:Ce(LuAG:Ce) single-crystal and transparent polycrystalline ceramics at a high temperature of 600 °C. The results showed that the CE^3+^ comprehensive RL strength of a single crystal increased by 1.4 times from RT-450 °C. Polycrystalline ceramics increased by 1.9 times from room temperature to 300 °C, i.e., LuAG:Ce scintillators can be used effectively over a wide temperature range. Bartosiewicz et al. [120] studied the growth process, crystal composition, and optical and scintillation properties of the Lu_3_Al_5_O_12_:La (La = 0–0.45%) single crystal. They found that La doping significantly reduced the flashing afterglow of the LuAG:La crystal and increased the flashing output. Representative studies on transparent scintillating ceramics from recent years are summarized in Table 4.

Compared with those from a single crystal, LuAG-based scintillating ceramics have the advantages of low preparation temperature, simple process, and low cost, and have important application prospects and development potential. Many scholars have continuously advanced their research work on the preparation, processing technology, and parameters of Lu_3_Al_5_O_12_(LuAG) doped with luminescent ions to provide an important reference basis for the design and preparation of new components of LuAG-based scintillating ceramics. At present, rare earth ion doping (Ce^3+^, Pr^3+^, etc.) is a kind of scintillating material with active research. This kind of material is mainly prepared by the precipitation method, and there is much research on its performance indexes (optical yield, energy resolution, radiation damage resistance, etc.). However, the amount of sinter used in the preparation of transparent ceramics and the amount of doped luminous ions are seldom studied, which should be the focus of future research work.

### 5.3. Summary

With the in-depth research on transparent nano-ceramics, electro-optic and scintillation transparent nano-ceramics have also been found to have great application prospects. The hot-pressing two-step approach and sol–gel spin-coating method are two commonly utilized preparation procedures for the former. The compactness of ceramic materials can be considerably improved by sintering mixed oxide during the preparation process. The temperature and electric field strength, for example, have an effect on the transmittance of optoceramics, according to the current study. However, one of the next important research topics will be how to manage these parameters to change the transmittance of optoceramic materials. Scintillating transparent nano-ceramics, on the other hand, have a wide range of applications in the medical field, physics, and industry due to their scintillation function. The majority of scintillation transparent ceramics research now focuses on the preparation method, powder size, material characteristics, and so on. Following a review of the literature, it was discovered that the majority of scintillation transparent ceramics are made using the co-precipitation process, with scintillation qualities increasing via doping with scintillation ions (mostly rare earth ions Ce^3+^, Pr^3+^, etc.). Furthermore, the specific application of scintillation transparent ceramics and how to improve the transparency of ceramics (through a reasonable sintering method) and light output (through the doping of luminescent ions) will be the research direction and technical difficulties of the future.

## 6. Discussion

### 6.1. Effect of the Particle Diameter on Fabrication

**Figure 18:**
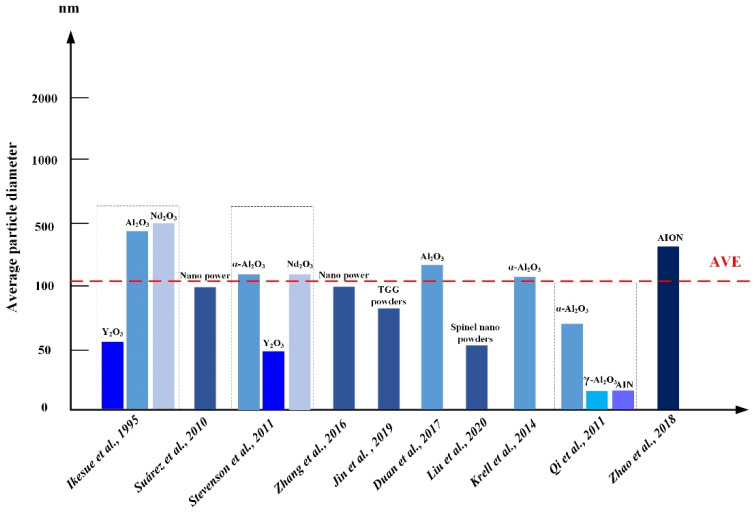
Average diameter of nano powders in the reviewed transparent nano-ceramics [12,14,15,17,43,52,81,83,86,88].

Figure 18 shows the average particle diameter of nano powders in the reviewed transparent nano-ceramics. As depicted in Figure 18, the average particle diameter ranges from 50 to 1000 nm, and the average is close to 150 nm. At present, most of the existing powders are of nano scale. Further reducing the particle diameter means that the manufacturing cost of powder is increased. For example, by laser reprocessing, the particle diameter of commercial powder can be further reduced, resulting in the desired specific surface areas. This is because polycrystalline transparent ceramics need to reach a relative density of more than 99.9%; that is, the porosity should be less than 1/10,000. It is difficult to eliminate these micropores via the conventional sintering process because the pores trapped in the crystal during grain growth are very difficult to discharge by diffusion at the end of sintering. Therefore, it is necessary to use ultra-fine nano powder with high purity and high activity and to adopt a multistage sintering process or sintering for a long time under low temperatures and a vacuum to eliminate pores.

### 6.2. Relationship between Nano Powders and Performance

According to the types of materials, transparent nano-ceramic materials are mainly divided into metal oxides and non-metal oxides [2]. Transparent nano-ceramics based on metal oxide materials mainly include those based on alumina (Al_2_O_3_), magnesia (MgO), zirconia (ZrO_2_), yttria (Y_2_O_3_), lutetia (Lu_2_O_3_), and other oxides. Transparent nano-ceramics based on non-metal oxide materials mainly include those based on AlON and AlN, sialon and silicon nitride (Si_3_N_4_), and fluoride. To meet the needs of different scenarios, the required nano-transparent ceramic functional materials can be prepared by selecting different nano powder. For example, in order to solve lighting applications, alumina (Al_2_O_3_)-based nano-powders can be selected for sintering. To meet the requirements of the gain medium of high-powered lasers, doped YAG transparent nano-ceramics are generally selected. Therefore, by selecting different types of nano powders for preparation, we can obtain different types of transparent nano-ceramics. For example, some need to have high-temperature resistance, and some need to have a magneto-optical effect. Judging from the existing research on transparent nano-ceramics, those based on alumina (Al_2_O_3_) are popular research topics and have also been used in commercial applications that are closely related to the chemical and physical properties of Al. In addition, the doping of rare earth elements can significantly improve the performance of transparent nano-ceramics. The most typical example is the doping of rare earth elements in YAG transparent nano-ceramics, which can significantly improve their performance. Additionally, the Verdet constant of TAG transparent nano-ceramics can be changed by adding rare earth elements.

### 6.3. Comparison of Different Sintering Methods

At present, the main sintering methods for preparing transparent nano-ceramics include HP, vacuum sintering, HIP, SPS, and microwave sintering. HP sintering is a high-pressure, low-strain-rate powder metallurgy process in which the creep process of sintering is controlled by applying heat and pressure. Due to the simultaneous application of force and heat, transparent nano-ceramics can thus be prepared at relatively low temperatures and achieve the desired density. High pressure can inhibit grain growth and induce plastic deformation to eliminate pores in grains, so the sintering mechanism under high pressure is completely different from that under normal pressure. Vacuum sintering belongs to the method of pressureless sintering. In a vacuum environment, a pressure difference is formed between the inside of the ceramic and the outside world, which helps the discharge of pores, reduces the porosity of the ceramic, makes the grains grow, and forms a high-density ceramic material. Vacuum sintering has the advantages of simple operation, low cost, and high production efficiency. It is currently the most widely used transparent ceramic sintering technology. Some oxide-based ceramic materials, such as rare-earth-doped YAG, Y_2_O_3_ and Al_2_O_3_, and other transparent nano-ceramic ceramic materials, can be prepared by vacuum sintering. For the traditional sintering process, HIP is a key step in the preparation of transparent nano-ceramics with high light transmittance, which can reduce the porosity inside the material and thus maximize the material density. To reduce manufacturing costs, HIP is usually used as the last step in the two-step sintering methods (HP + HIP sintering; vacuum sintering + HIP sintering).

The SPS method is a new technique for heating and sintering by directly passing a pulse current between the nano-powder particles. Compared with the HIP method or the HP method, it has the distinctive features of a fast heating rate, short sintering time, controllable structure, energy savings, and environmental protection. In addition, since the sintering of transparent nano-ceramic materials requires the consideration of various factors, the SPS method can also be easily combined with optimization, such as doping of sintering aids, which makes its application range larger. During the microwave-sintering process, the processing material heats up very quickly, which can be carried out at low sintering temperatures and short sintering times to obtain nano-ceramics with high transparency. In contrast, microwave sintering has the advantages of a short fabrication time and low processing cost.

### 6.4. Relationship between Nano Ceramic Materials and Biomedicine

Nano-ceramics have been successfully employed in medical diagnoses (nano biosensors, nano bioimaging) and medical therapy (nano drug loading, nano biomedical materials, nano biocompatible organs) due to their unique sensing and biological properties. Especially, nanoceramics have great application prospects in the manufacture of biocompatible organs (artificial organs, artificial blood vessels, and artificial bones) [121]. Abe et al. [122] evaluated the biocompatibility of several nano-ceramic particles (TiO_2_, In_2_O_3_, ITO, Y_2_O_3_: Eu, and CuO) with bone cells, tumor cells, and hepatocytes. The results showed that the nanoparticles could be safely used in industrial and biomedical applications. Manonmani et al. [123] found that a novel nano triphasic bioceramic composite could effectively improve the corrosion resistance and surface cell activity of orthopedic implants. Traditional medical materials and artificial organs and tissues made of various conventional materials have limited compatibility with the patient’s body in clinical use, so it is difficult to fundamentally solve the disease in the patient’s body. Nano-scale materials have good biocompatibility, which means they fit the structure of cells in the human body to a high degree and can successfully avoid problems such as postoperative trauma and infection [124]. At present, transparent nano-ceramics biocompatibility research and development is still limited, and there is still a long way to go before they are widely used in clinical practice. Furthermore, nanoparticles’ high compatibility and degradability with blood tissue will be a long-term study focus in the future.

## 7. Outlook

Transparent nano-ceramics are a new class of materials, and various preparation strategies have been developed, especially using nano-powders, to obtain them with various compositions and properties to meet the needs of different applications. Here, we reviewed the research progress and potential applications of mainstream transparent nano-ceramics. In the future, the development of transparent nano-ceramics and their potential applications are anticipated as follows:

(1) For the preparation of transparent nano-ceramics, high-purity and high-quality nano powder is very important, especially the average particle diameter of nano powder. At present, in the existing literature, many studies use the nano powder of commercial companies to prepare them, and the average particle diameter ranges from 50 to 100 nm (the average of 150 nm). However, in order to prepare high-quality transparent nano-ceramics, it is necessary to pretreat the existing nano powders, such as further improving the sintering activity of nano powders and reducing the average diameter of particles through laser processing or other methods, so that the light transmittance can be improved after the subsequent fabricating process.
(2) The preparation of transparent nano-ceramics via new sintering technologies, such as spark plasma sintering and laser sintering, is still in the exploratory stage. The existing research on these sintering technologies is mainly based on experiments, and there is a lack of theoretical research to clarify the action mechanism of micro defects in the sintering process, especially to establish the thermodynamics and kinetics of sintering reaction. In addition, with the background of carbon peak and carbon neutralization, reducing the sintering temperature and sintering in a lower temperature range to obtain transparent nano-ceramics with excellent properties is still an important research direction in the future.
(3) The preparation of transparent nano-ceramics via new sintering technologies, such as spark plasma sintering and laser sintering, is still in the exploratory stage. New sintering processes, such as spark plasma sintering and laser sintering, are continuously being investigated for the preparation of transparent nano-ceramics. Especially, the laser-induced plasma process, with the advantages of high-power plasma and an ultrafast laser, has potential for the fabrication of transparent nano-ceramics [125]. However, there is a shortage of theoretical studies to explain the action mechanism of micro defects in the sintering process and especially to establish the thermodynamics and kinetics of the sintering reaction in current studies. Furthermore, in consideration of the carbon peak and carbon neutralization, lowering the sintering temperature and sintering in a lower temperature range to create transparent nano-ceramics with good characteristics appears to be an important research direction.
(4) Transmittance is one of the most important indicators for transparent nano-ceramics, and the microstructure is the most important component determining it. Transparent nano-ceramic samples supplemented with sintering aids have low porosity, allowing the grains to tightly fill the space and achieve an excellent microstructure. Different varieties of transparent nano-ceramics, in general, need different sintering aids. Furthermore, the doping ratio of sintering aids impacts the sintering process, affecting the transmittance for the same type of transparent ceramics. In the future, not only will trials be used to determine the best sintering aid, but computer simulations will also be used to save time and cost.
(5) The powder particle size, sintering temperature, sintering duration, sintering environment, sintering pressure, and sintering aids are the primary elements impacting the sintering of transparent nano-ceramics. Aside from the standard light transmittance and physical properties, the indicators for evaluating transparent nano-ceramics must also take into account the manufacturing cost and environmental impact. As a result, sintering transparent nano-ceramics is an MIMO (multi-input, multi-output) process [126,127]. Multi-objective optimization of its preparation process is necessary for the future, which will help to lower its production costs and environmental impact.
(6) Finally, theoretical research on transparent nano-ceramics is still in its early stages. For example, molecular dynamic models are efficient tools for studying the mechanism of action at the microscopic scale [128,129], which have been used to describe the microstructures of transparent glass ceramics [130,131]. As a result, researchers must develop suitable theoretical models to guide and optimize the transparent nano-ceramic preparation process. Then, the development of transparent nano-ceramic technology may be more promising if theoretical analysis and experimental results are combined.

## Figures and Tables

**Table 1:** Summary of doped YAG transparent nano-ceramics described in the text grouped by doped type and published year.

Doping Type	Year, Powder, and Fabrication	Findings	Performance	Remarks
Nd^3+^-doped	1995; the starting nano powders include Y_2_O_3_ (60 nm), Al_2_O_3_ (400 nm), and Nd_2_O_3_ (500 nm); solid-state reaction method (Czochralski method).	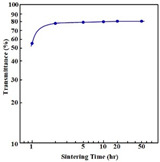 The average grain size and relative density of the 1.1 at. % Nd:YAG ceramics were about 50 μm and 99.98%, respectively.	The optical scattering loss of Nd:YAG was about 0.9%/cm. Oscillation threshold of 309 mW and a slope efficiency of 28% [12].	For the first time, polycrystalline ceramics were successfully used for effective laser cutting.
	2002; raw nano powder of oxide of aluminum, yttrium, and neodymium; ball milling -> slip casting -> vacuum sintering -> YAG transparent nano-ceramics.	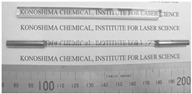 The pore volume concentration was 1 ppm, and the average diameter of particles was about 10 μm. The grain boundary width was only about 1 nm [13].	In laser experiment of Nd:YAG ceramic and single-crystal rods, the output powers of 88 W and 99 W were obtained, respectively [13].	Compared with single-crystal Nd:YAG, the light-to-light efficiency of Nd:YAG nanocrystalline ceramics needs to be further improved.
	2010; an average particle size of 100 nm by reverse-strike precipitation method; HIP method.	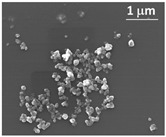 Freeze drying was proven to be an effective method to avoid caking and produced a material nano size distribution with uniform particles [14].	The infrared transmittance of the sample was 80%, and its emission spectrum was the same as 1 at.% Nd:YAG single crystal [14].	Nd: YAG nano powder with an average size of about 100 nm was prepared for the first time.
	2021; high-purity powder mixture; cold isostatic pressing.	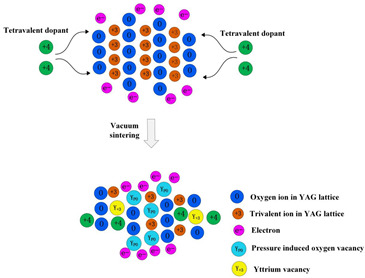 The addition of TEOS promoted the densification of transparent ceramics.	The transmittance of the 0.5 wt.% TEOS sample reached 75% in the near-infrared region [18].	The densification rate of Nd: YAG transparent ceramics could be adjusted by adding different wt.% TEOS so as to improve its transmittance.
Ho-doped	2015; the nano powders were made up of near-spherical particles; solid-state reaction involving a pre-calcining stage.	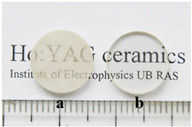 The transmittance in the infrared region was 82% [21].	The slope efficiency of laser oscillations in the fabricated Ho:YAG transparent ceramic sample for pumping power was 40% (at 1.85 μm) [21].	Based on the nano-powders prepared by laser ablation, Ho:YAG optical ceramics with finer particle size were prepared.
	2018; uniform ceramic grain; HIP method.	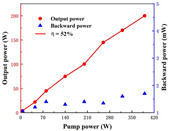 The total absorption spectral width was about 16 nm and suitable for pumping of diode lasers or fiber lasers, and the light-to-light efficiency was 52%.	The in-band pumping method produced a 2117 nm laser with an output power of 24.6 W [22].	Further development of large-scale, YAG transparent ceramics with low Ho^3+^ doping concentration is required to alleviate the thermal effect during the lasing process.
Er-doped	2018; high-purity 0.5 at.% Er^3+^:YAG powder; SPS+HIP methods.	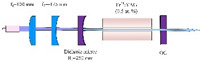 At 400 and 1100 nm wavelengths, the on-line transmission values were 75.8% and 82.7%, respectively [25].	The light–light efficiency of laser was 20%, and the maximum slope efficiency was 31% [25].	Transmission values of the Er^3+^: YAG transparent ceramics were lower than that of Er^3+^:YAG single crystals, which requires further improvement of the fabrication process.
Tm-doped	2010.	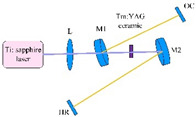 Light-to-light efficiency was 22%.	Under an absorbed pump power of 2.21 W at 785 nm, an output power of up to 860 mW was produced [29].	Tm:YAG ceramic is a promising laser working medium. Higher power and efficiency can be achieved by using an improved laser cavity and an optimized transmission optical path.
Yb-doped	2008.	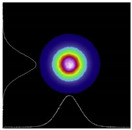 The transverse intensity distribution of the Yb:YAG ceramic laser beam was a Gaussian beam [31].	The ceramic Yb:YAG laser exhibited a continuous tunability at a maximum output power of 1.6 W [31].	Except for crystal Yb:YAG investigations, this was the first study of the tunability of ceramic Yb:YAG lasers.

**Table 2:** Comparison of characteristics of Faraday isolators with different magneto-optical media [38,56]. Reprinted with permission from Ref. [38]. Copyright 2018, copyright CNKI. Reprinted with permission from Ref. [56]. Copyright 2017, copyright OSA.

Medium	Isolation Ratio@ Laser Power	Isolation Ratio@ Laser Power	Water Cooling
TGG crystal	30 dB@650 W	6.5 m@340 W	Optional
TGG transparent nano-ceramic	30 dB@340 W	6.5 m@340 W	Optional
TAG transparent nano-ceramic	38 dB@300 W	8 m@300 W	Required
Ce: TAG transparent nano-ceramic (0.1 at.%)	31 dB@300 W	3.8 m@300 W	Required

**Table 3:** Summary of electro-optical transparent nano-ceramics described in the text grouped by publication year.

Year, Authors	Powder and Fabrication	Findings	Performance	Remarks
1970, Haertling et al. [102]	Preparation of optoceramics with a thickness of 1 mm using a two-step hot-pressing method.	The density reached up to 99% of the standard density and the transmittance reached up to 80%.	When the wavelength increased, the light transmission performance also gradually increased to 80%.	The hot-pressing firing method could improve the density of ceramics, but the light transmission performance needed to be improved.
2016, Somwan et al. [103]	Mixture of Bi_2_O_3_ and CuO; vibrating grinding and sintering.	After adding mixed oxides, the sintering temperature decreased by nearly 50 °C to 1200 °C.	At 1200 °C, the induced strain of the enhanced electric field reached 0.0079%.	Higher dielectric constants could be obtained at lower sintering temperatures.
2017, Zhang et al. [105]	Mixture of 3% lanthanum, 75% PMN, and 25% PT; two-step hot-pressing method.	As the temperature increased, the half-wave voltage increased from 200 to 400 V.	The electro-optic coefficient increased with an increase in temperature.	The ferroelectric preparation process and transmittance could be controlled by temperature.
2018, Samanta et al. [104]	Mixture of 69 ppm Fe^3+^ and 78 ppm Nb^5+^; sol–gel.	Conductivity increased by two orders of magnitude as the sample changed from 100 Hz to 1 MHz.	Conductivity was proportional to frequency.	The conductivity could be controlled by controlling the magnitude of the frequency.
2018, Wang et al. [106]	PMN-PT/CFO thin films; sol–gel spin coating.	The temperature rose from 650 to 730 degrees Celsius; the leakage current decreased from 97.54 to 40.59.	The ferroelectric properties decreased with an increase in the ratio of CFO to PMN-PT and increased with an increase in temperature.	The coupling effect between the ferroelectric phase and the ferromagnetic phase was observed, which will pave the way for the preparation of multifunctional crystals in the future.
2021, Ze et al. [97]	PMN-PT ceramic materials doped with Sm; two-step sintering method.	When the Sm doping amount increased from 0 to 2%, the PMN-PT decreased from 3.15 to 3.05 eV.	When there was Sm doping, it affected the size of the electro-optic coefficient.	The optical power could be controlled by doping Sm.

**Table 4:** Summary of scintillation transparent nano-ceramics described in the text grouped by publication year.

Year, Authors	Powder and Fabrication	Findings	Performance	Remarks
2017, Zhou et al. [115]	99.9% strontium nitrate hydrate, 99.9% neodymium nitrate hydrate, and 99.9% potassium fluoride hydrate; the chemical precipitation method.	SrF_2_ nanoparticles with Nd^3+^ doping concentrations up to 2 mol% kept a single cubic fluoride structure.	The synthesized powder could prepare transparent ceramics with a transmittance of about 80% at 1060 nm.	Nd^3+^ was successfully introduced into the SrF_2_ lattice, making it possible to use this material to make transparent ceramics.
2018, Yi et al. [116]	Nd:(Ca_0.94_Gd_0.06_)_2.06_ nano powder; deionized water coprecipitation	As the Nd^3+^ content increased from 0.5 to 5.0, the measured lifetime dropped sharply from 484.9 μs to 47.8 μs.	The transparent ceramic had high transparency and an almost non-porous microstructure.	The thermal conductivity of Nd:(Ca_0.94_Gd_0.06_)F_2.06_ transparent ceramics was better than that of traditional laser glass, and transparent ceramic is a promising laser material.
2019, Hostaša et al. [118]	Industrial oxide powder, 0.3% Ce: GGAG,Ce_0.009_Gd_2.991_Al_2_Ga_3_O_12_ ceramic sample.	At 1250 °C, the formation of the GGAG phase could be observed. This corresponds to the increase in shrinkage observed above 1200 °C, with the optimum at 1430 °C.	TEOS was determined to be the most suitable sintering aid in the tests, providing the highest sample density and clarity.	The optimum amount of sintering aid and the corresponding sintering cycle should be further investigated.
2020, Trofimov et al. [119]	High-purity (99.99%) starting material and 0.5% tetraethyl orthosilicate (TEOS); the coprecipitation method.	The Ce^3+^ comprehensive RL strength of single crystal increased 1.4 times from RT-450 °C, while polycrystalline ceramics increased 1.9 times from room temperature to 300 °C.	Both single-crystal and polycrystalline ceramics exhibited high optical transparency up to about 2.5 eV.	The LuAG:Ce scintillator could adapt to a wide temperature range and could be applied to many occasions.
2020, Bartosiewicz et al. [120]	Mixure of 4 N-purity Lu_2_O_3_, Al_2_O_3_, and La_2_O_3_ oxides; the μ-PD method using RF induction heating.	With the increase in La content, the main luminescence in the UV region gradually moved from 330 nm to 295 nm.	Lu_3_Al_5_O_12_ produced strong luminescence in the deep ultraviolet spectral range	LuAG: La doping significantly reduced the scintillation afterglow of LuAG: La crystals. Therefore, it is possible to generate new scintillators in the deep ultraviolet range.

## Data Availability

Not applicable.

## References

[1] Wang S., Zhang J., Luo D., Gu G., Tang D., Dong Z., Tan G., Que W., Zhang T., Li S. (2013). Transparent ceramics: Processing, materials and applications. Prog. Solid State Chem..

[2] Xiao Z., Yu S., Li Y., Ruan S., Kong L., Huang Q., Huang Z., Zhou K., Su H., Yao Z. (2020). Materials development and potential applications of transparent ceramics: A review. Mater. Sci. Eng. R Rep..

[3] Pradhan A., Zhang K., Loutts G. (2004). Synthesis of neodymium added yttrium aluminum garnet (YAG) nanocrystalline powders leading to transparent ceramics. Mater. Res. Bull..

[4] Niihara K. (1991). New design concept of structural ceramics-ceramic nanocomposites. J. Ceram. Soc. Jpn..

[5] Goldstein A., Krell A., Kleebe A. (2016). Transparent ceramics at 50: Progress made and further prospects. J. Am. Ceram. Soc..

[6] Merac M.R., Kleebe H.J., Müller M.M., Reimanis I.E. (2013). Fifty years of research and development coming to fruition; unraveling the complex interactions during processing of transparent magnesium aluminate (MgAl_2_O_4_) spinel. J. Am. Ceram. Soc..

[7] Cheng J., Dinesh A., Zhang Y., Rustum R. (2002). Microwave sintering of transparent alumina. Mater. Lett..

[8] Li S. (2007). Specialty Ceramic Technology. J. Wuhan Univ. Technol..

[9] Drozdowski W., Brylew K., Witkowski M.E., Drewniak A., Masewicz A., Wojtowica A.J., Kisielewski J., Swirkowicz M. (2016). Effect of Lu-to-Y ratio and Mo coactivationon scintillation properties of LuYAG:Pr and LuAG:Pr,Mo crystals. Opt. Mater..

[10] Jiang Y., Liu Q., Mao X., Su S., Li X., Liu X., Feng Y., Chen X., Li J. (2020). Influence of CaO on microstructure and properties of MgAl_2_O_4_ transparent ceramics. Opt. Mater..

[11] Liu X., Chen F., Zhang F., Zhang H., Zhang Z., Wang J., Wang S., Huang Z. (2013). Hard transparent AlON ceramic for visible/IR windows. Int. J. Refract. Met. Hard Mater..

[12] Ikesue A., Kinoshita T., Kamata K., Yoshida K. (1995). Fabrication and Optical Properties of High-Performance Polycrystalline Nd:YAG Ceramics for Solid-State Lasers. J. Am. Ceram. Soc..

[13] Lu J., Ueda K., Yagi H., Yanagitani T., Akiyama Y., Kaminskii A.A., Alloys J. (2002). Neodymium doped yttrium aluminum garnet (Y_3_Al_5_O_12_) nanocrystalline ceramics-a new generation of solid state laser and optical materials. J. Alloy. Compd..

[14] Suárez M., Fernández A., Menéndez J., Nygren M., Torrecillas R., Zhao Z. (2010). Hot isostatic pressing of optically active Nd:YAG powders doped by a colloidal processing route. J. Eur. Ceram. Soc..

[15] Stevenson A., Kupp E., Messing G. (2011). Low temperature, transient liquid phase sintering of B_2_O_3_-SiO_2_-doped Nd:YAG transparent ceramics. J. Mater. Res..

[16] Yavetskiy R., Baumer V., Doroshenko A., Kopylov Y., Kosyanova D., Kravchenkoc V., Parkhomenkoa S., Tolmacheva A. (2014). Phase formation and densification peculiarities of Y_3_Al_5_O_12_:Nd^3+^ during reactive sintering. J. Cryst. Growth.

[17] Zhang X., Fan G., Lu W., Chen Y., Ruan X. (2016). Effect of the spark plasma sintering parameters, LiF additive, and Nd dopant on the microwave dielectric and optical properties of transparent YAG ceramics. J. Eur. Ceram. Soc..

[18] Jia W., Wei Q., Zhang H., Su C., Ren G., Zhao M., Ma C. (2021). Comparative analyses of the influence of TEOS additives on the sintering kinetics of Nd: YAG transparent ceramics. J. Mater. Sci. Mater. Electron..

[19] Zhang W., Zhou J., Liu W., Li J., Wang L., Jiang B., Pan Y., Cheng X., Xu J. (2010). Fabrication, properties and laser performance of Ho:YAG transparent ceramic. J. Alloy. Compd..

[20] Yang H., Zhang J., Qin X., Luo D., Ma J., Tang D., Chen H., Shen D., Zhang Q. (2012). Polycrystalline Ho:YAG transparent ceramics for eye-safe solid state laser applications. J. Am. Ceram. Soc..

[21] Bagayev S., Osipov V., Vatnik S., Shitov V., Vedin I., Platonov V., Steinberg I., Maksimov R. (2015). Ho:YAG transparent ceramics based on nanopowders produced by laser ablation method: Fabrication, optical properties, and laser performance. Opt. Mater..

[22] Zhao Y., Wang J., Yao W., Shao Z., Shen C., Yin D., Wang Y., Liu P., Zhou W., Tang D. (2018). High power Ho-doped sesquioxide ceramic laser in-band pumped by a Tm-doped all-fiber MOPA. IEEE Photon. J..

[23] Zhang C., Shen D., Wang Y., Qian L., Zhang J., Qin X., Tang D., Yang X., Zhao T. (2011). High-power polycrystalline Er:YAG ceramic laser at 1617 nm. Opt. Lett..

[24] Zhang X., Shen D., Huang H., Liu J., Zhang J., Tang D., Fan D. (2015). Passively Q-switched 1617-nm polycrystalline ceramic Er:YAG laser using a Cr:ZnSe saturable absorber. Appl. Phys. B.

[25] Bigotta S., Galecki L., Katz A., Bohmler J., Lemonnier S., Barraud E., Leriche A., Eichhorn M. (2018). Resonantly pumped eye-safe Er^3+^:YAG SPS-HIP ceramic laser. Opt. Express.

[26] Zou Y., Wei Z., Wang Q., Zhan M., Li D., Zhang Z., Zhang J., Tang D. (2013). High-efficiency diode-pumped Tm:YAG ceramic laser. Opt. Mater..

[27] Liu P., Jin L., Liu X., Huang H., Zhang J., Tang D., Shen D. (2016). A diode-pumped dual-wavelength Tm, Ho: YAG ceramic laser. IEEE Photon. J..

[28] Zhang W., Pan Y., Zhou J., Liu W., Li J., Jiang B., Cheng X., Xu J. (2009). Diode-pumped Tm:YAG ceramic laser. J. Am. Ceram. Soc..

[29] Zou Y., Zhang Y., Zhong X., Wei Z., Zhang W., Jiang B., Pan Y. (2010). Efficient Tm:YAG ceramic laser at 2 μm. Chin. Phys. Lett..

[30] Zhan M., Zou Y., Lin Q., Wang Z., Han H., Lu L., Wei Z., Zhang J., Tang D. (2014). Ti:saphire pumped passively mode-locked Tm:YAG ceramic laser. Acta Phys. Sin..

[31] Nakamura S., Yoshioka H., Matsubara Y., Ogawa T., Wada S. (2008). Efficient tunable Yb:YAG ceramic laser. Opt. Commun..

[32] Luo D., Jian Z., Xu C., Qin X., Tang D., Ma J. (2012). Fabrication and laser properties of transparent Yb:YAG ceramics. Opt. Mater..

[33] Sanghera J., Kim W., Guillermo V., Brandon S., Colin B. (2012). Ceramic laser materials. Opt. Mater..

[34] Mandl A., Klimek D. (2010). Textron’s J-HPSSL 100 kW ThinZag laser program. Conference on Lasers and Electro-Optics.

[35] Yuan J., Duan X., Yao B., Li J., Cui Z., Shen Y., Dai T., Ju Y., Li C., Kou H. (2015). Dual-end-pumped high-power Cr^2+^:ZnS passively Q-switched Ho:YAG ceramic laser. Appl. Phys..

[36] Feizbakhsh M., Yazd N.S., Keshavarzi A., Doosti A. (2022). Anisotropic crystallization of YAG on the surface of glass by CO_2_ laser irradiation. Crystengcomm.

[37] Li T., Zhou T., Cao Y., Cai Z., Zhao C., Yuan M., Zheng X., Huang G., Wang Z., Zhang L. (2022). Optical properties and energy transfer performances in high quality Cr,Nd:YAG transparent laser ceramics for solar pumped lasers. Opt. Express.

[38] Li J., Dai J., Pan Y. (2018). Research progress on magneto-optical transparent ceramics. J. Inorg. Mater..

[39] Yoshida H., Tsubakimoto K., Fujimoto Y., Mikami K., Kinoshita H. (2011). Optical properties and Faraday effect of ceramic terbium gallium garnet for a room temperature Faraday rotator. Opt. Express.

[40] Wu Y., Sun Z., Feng G., Wang S., Xu L., Wu S. (2021). Preparation and properties of novel Tb_3_Sc_2_Al_3_O_12_ (TSAG) magneto-optical transparent ceramic. J. Eur. Ceram. Soc..

[41] Khazanov E. (2003). Investigation of faraday isolator and faraday mirror designs for multi-kilowatt power lasers. High-Power Lasers Appl..

[42] Yasuhara R., Tokita S., Kawanaka J., Kawashima T., Kan H., Yagi H., Nozawa H., Yanagitani T., Fujimoto Y., Yoshida H. (2007). Cryogenic temperature characteristics of Verdet constant on terbium gallium garnet ceramics. Opt. Express.

[43] Jin W., Gai L., Chen J., Lin H., Li C., Su L., Wu A., Zeng F. (2019). Fabrication and magneto-optical properties of TGG transparent ceramics. Phys. B.

[44] Li X., Snetkov I., Yakovlev A., Liu Q., Liu X., Liu Z., Chen P., Zhu D., Wu L., Yang Z. (2021). Fabrication and performance evaluation of novel transparent ceramics RE:Tb_3_Ga_5_O_12_ (RE = Pr, Tm, Dy) toward magneto-optical application. J. Adv. Ceram..

[45] Moriana A.D., Zhang S. (2022). Determining the effects of BaTiO_3_ template alignment on template grain growth of Pb(Mg_1/3_Nb_2/3_)O_3_ -PbTiO_3_ and effects on piezoelectric properties. J. Eur. Ceram. Soc..

[46] Zhang H., Gao Y., Huang C., Chen Y., Dou R., Zhang Q. (2021). Electronic structure, optical dispersion and luminescence properties of terbium gallium garnet crystal. Crystengcomm.

[47] Li X., Liu Q., Liu X., Chen X., Wu L., Xie T., Shi Y., Chen H., Li J. (2019). Novel (Tb_0.99_Ce_0.01_)_3_Ga_5_O_12_ magneto-optical ceramics for Faraday isolators. Scr. Mater..

[48] Yasuhara R., Snetkov I., Starobor A., Zheleznov D., Palashov O., Khazanov E., Nozawa H., Yanagitani T. (2014). Terbium gallium garnet ceramic Faraday rotator for high-power laser application. Opt. Lett..

[49] Lin H., Zhou S., Teng H. (2011). Synthesis of Tb_3_Al_5_O_12_ (TAG) transparent ceramics for potential magneto-optical applications. Opt. Mater..

[50] Kaminskii A., Eichler H., Reiche P., Uecker R. (2005). SRS risk potential in Faraday rotator Tb_3_Ga_5_O_12_ crystals for high-peak power lasers. Laser Phys. Lett..

[51] Chen C., Yi X., Zhang S., Feng Y., Tang Y., Lin H., Zhou S. (2015). Vacuum sintering of Tb_3_Al_5_O_12_ transparent ceramics with combined TEOS+MgO sintering aids. Ceram. Int..

[52] Duan P., Liu P., Xu X., Wang W., Wan Z., Zhang S., Wang Y., Zhang J. (2017). Fabrication of transparent Tb_3_Al_5_O_12_ ceramics by hot isostatic pressing sintering. J. Am. Ceram. Soc..

[53] Chen C., Zhou S., Lin H., Yi Q. (2012). Fabrication and performance optimization of the magneto-optical (Tb_1-x_Rx)_3_Al_5_O_12_ (R = Y, Ce) transparent ceramics. Appl. Phys. Lett..

[54] Chen C., Ni Y., Zhou S., Hui L., Yi X. (2013). Preparation of (Tb_0.8_Y_0.2_)_3_Al_5_O_12_ transparent ceramic as novel magneto-optical isolator material. Chin. Opt. Lett..

[55] Snetkov I., Permin D., Balabanov S., Palashov O. (2016). Wavelength dependence of Verdet constant of Tb^3+^: Y_2_O_3_ ceramics. Appl. Phys. Lett..

[56] Furuse H., Yasuhara R. (2017). Magneto-optical characteristics of holmium oxide (Ho_2_O_3_) ceramics. Opt. Mater. Express.

[57] Morales J., Amos N., Khizroev S., Garay J. (2011). Magneto-optical Faraday effect in nanocrystalline oxides. J. Appl. Phys..

[58] Hu D., Li X., Snetkov I., Yakovlev A., Balabanov S., Ivanov M., Liu X., Liu Z., Tian F., Xie T. (2021). Fabrication, microstructure and optical characterizations of holmium oxide (Ho_2_O_3_) transparent ceramics. J. Non-Cryst. Solids.

[59] Furuse H., Yasuhara R., Hiraga K., Zhou S. (2016). High Verdet constant of Ti-doped terbium aluminum garnet (TAG) ceramics. Opt. Mater. Express.

[60] Starobor A.V., Kuznetsov I.K., Palashov O.V., Pestov A.E., Chkhalo N.I. (2021). Faraday Isolator with composite magneto-optical TGG-sapphire elements. IEEE J. Quantum Electron..

[61] Lin H., Jia H., Zhou L., Li N., Liu B., He J., Yao G., Li S., Zhou Y., Li C. (2022). Magneto-optical and fluorescence properties of Tb^3+^ doped glass-ceramics containing AlPO_4_. J. Non-Cryst. Solids.

[62] Zhang L., Li X., Hu D., Liu Z., Xie T., Wu L., Yang Z., Li J. (2021). Fabrication and properties of transparentmagneto-optical ceramics. J. Eur. Ceram. Soc..

[63] Shbool M., Al-Abdallat Y., Jawwad A.K.A., Alsharairi S., Abu-Ghannam L., Abu-Khajil E.E., Badwan A. (2021). Examining the effect of nano-additions of rare earth elements on the hardness of body armor ceramic. Indian J. Eng. Mater. S.

[64] Yang X., Lai Y., Zeng Y., Yang F., Huang F., Li B., Wang F., Wu C., Su H. (2022). Spinel-type solid solution ceramic MgAl_2_O_4_-Mg_2_TiO_4_ with excellent microwave dielectric properties. J. Alloy. Compd..

[65] Tobias B., Sergio Y.G., de Oliveira A.P.N., Travitzky N., Hotza D. (2017). Transparent ceramic and glass-ceramic materials for armor applications—ScienceDirect. Ceram. Int..

[66] Strassburger E. (2009). Ballistic testing of transparent armour ceramics. J. Eur. Ceram. Soc..

[67] Grujicic M., Bell W., Pandurangan B. (2012). Design and material selection guidelines and strategies for transparent armor systems. Mater. Des..

[68] Subhash G. (2013). Transparent Armor Materials. Exp. Mech..

[69] Salem J. (2019). Transparent armor ceramics as spacecraft windows transparent armor ceramics as spacecraft windows. J. Am. Ceram. Soc..

[70] Sepulveda J., Loutfy R., Chang S., Ibrahim S. (2011). High Performance Spinel Ceramics for IR Windows and Domes. Proc. Spie Int. Soc. Opt. Eng..

[71] Gilde G., Patel P., Sands J., Patterson P., Blodgett D., Duncan D., Hahn D. (2006). Evaluation of hot isostatic pressing parameters on the optical and ballistic properties of spinel for transparent armor. Am. Ceram. Soc..

[72] Bratton R. (1974). Translucent sintered MgAl_2_O_4_. J. Am. Ceram. Soc..

[73] Tsukuma K. (2006). Transparent MgAl_2_O_4_ spinel ceramics produced by HIP post-sintering. J. Ceram. Soc. Jpn..

[74] Villalobos G., Sanghera J., Aggarwal I. (2010). Degradation of magnesium aluminum spinel by lithium fluoride sintering aid. J. Am. Ceram. Soc..

[75] Esposito L., Piancastelli A., Martelli S. (2013). Production and characterization of transparent MgAl_2_O_4_ prepared by hot pressing. J. Eur. Ceram. Soc..

[76] Esposito L., Piancastelli A., Miceli P. (2015). A thermodynamic approach to obtaining transparent spinel (MgAl_2_O_4_) by hot pressing. J. Eur. Ceram. Soc..

[77] Boulesteixa R., Goldsteinb A., Perrièrea C., Maîtrea A., Katzb M., Coureauc C., Salléd C. (2021). Transparent ceramics green-microstructure optimization by pressure slip-casting: Cases of YAG and MgAl_2_O_4_. J. Eur. Ceram. Soc..

[78] Kagawa A. (2003). Effect of grain boundary microcracking on the light transmittance of sintered transparent MgAl_2_O_4_. J. Eur. Ceram. Soc..

[79] Zou Y., He D., Wei X., Yu R., Lu T., Chang X., Wang S., Li L. (2010). Nanosintering mechanism of MgAl_2_O_4_ transparent ceramics under high pressure. Mater. Chem. Phys..

[80] Meir S., Kalabukhov S., Froumin N., Dariel M., Frage N. (2010). Synthesis and densification of transparent magnesium aluminate spinel by SPS processing. J. Am. Ceram. Soc..

[81] Liu Q., Jing Y., Su S., Li X., Liu X., Feng Y., Chen X., Li J. (2020). Microstructure and properties of MgAl_2_O_4_ transparent ceramics fabricated by hot isostatic pressing. Opt. Mater..

[82] Liu Y., Zhu J., Dai B. (2020). Transparent MgAl_2_O_4_ ceramics prepared by microwave sintering and hot isostatic pressing—ScienceDirect. Ceram. Int..

[83] Krell A., Hutzler T., Klimke J. (2014). Defect strategies for an improved optical quality of transparent ceramics. Opt. Mater..

[84] Mccauley J.W., Corbin N.D. (1983). High temperature reactions and microstructures in the Al_2_O_3_-AlN system. Progress in Nitrogen Ceramics.

[85] Yamaguchi G., Yanagida H. (1959). Study on the reductive Spinel-A new spinel formula AlN–Al_2_O_3_ instead of the previous one Al_3_O_4_. Bull. Chem. Soc. Jpn..

[86] Qi J., Wang Y., Lu T., Yu Y., Pan L., Wei N., Wang J. (2011). Preparation and light transmission properties of AlON ceramics by the two-step method with nanosized Al_2_O_3_ and AlN. Metall. Mater. Trans. A.

[87] Jin X., Gao L., Sun J., Liu Y., Gui L. (2012). Highly transparent AlON pressurelessly sintered from powder synthesized by a novel carbothermal nitridation method. J. Am. Ceram. Soc..

[88] Zhao F., Qi J., Huang X., Guo X., Yu Y., Cao X., Wang Y., Wu D., Meng C., Lu T. (2018). Planetary ball-milling of AlON powder for highly transparent ceramics. J. Am. Ceram. Soc..

[89] Zhang Y., Wu H., Jia B., Zhang D., Wang Y., Zhang Z., Qu X., Qin M. (2022). Transparent AlON ceramics by nitriding combustion synthesis precursors and pressureless sintering method. Ceram. Int..

[90] Yuan X., Zhang F., Liu X., Zhang Z., Wang S. (2011). Fabrication of transparent AlON ceramics by solid-state reaction sintering. J. Inorg. Mater..

[91] Shan Y., Sun X., Ren B., Wu H., Wei X., Olevsky E.A., Xu J., Li J. (2018). Pressureless sintering of highly transparent AlON ceramics with CaCO_3_ doping. Scr. Mater..

[92] Li J., Zhang B., Tian R., Mao X., Zhang J., Wang S. (2021). Hot isostatic pressing of transparent AlON ceramics assisted by dissolution of gas inclusions. J. Eur. Ceram. Soc..

[93] Cheng X., Fu L., Su J., Liu W., Li Y., Mao P., Zhang L., Hui X. (2020). Research status and development trend of ALON ceramics. Mater. Rep..

[94] Mccauley J.W., Patel P., Chen M., Gilde G., Strassburger E., Paliwal B., Ramesh K.T., Dandekar D.P. (2009). AlON: A brief history of its emergence and evolution. J. Eur. Ceram. Soc..

[95] Goldman L.M., Smith M., Ramisetty M., Kashalikar U., Jha S., Sastri S. (2019). Scale up of large ALON windows. Window and Dome Technologies and Materials XVI.

[96] Liu L., Zhang C., Takahashia K., Nishimurac T., Segawad H., Hirosakia N., Xie R. (2019). Uniform and fine Mg-γ-AlON powders prepared from MgAl_2_O_4_: A promising precursor material for highly-transparent Mg-γ-AlON ceramics. J. Eur. Ceram. Soc..

[97] Ze F., Jiang X., Tian X., Zheng F., Cheng M., Zhao E., Ye W., Qin Y., Zhang Y. (2021). Ultratransparent PMN-PT electro-optic ceramics and its application in optical communication. Adv. Opt. Mater..

[98] Pramanika R., Sahukarb M.K., Mohanc Y., Praveenkumard B., Sangaward S.R., Arockiarajana A. (2019). Effect of grain size on piezoelectric, ferroelectric and dielectric properties of PMN-PT ceramics. Ceram. Int..

[99] Slodczyk A., Colomban P. (2010). Probing the nanodomain origin and phase transition mechanisms in (Un)Poled PMN-PT single crystals and textured ceramics. Materials.

[100] Lu G. (2013). Exploration of Optical Energy Storage and Nir Wavelength Conversion in Rare Earth Doped Relaxation Ceramics.

[101] Wei Z. (2014). Optical characteristics of Er^3+^-doped PMN-PT transparent ceramics. Ceram. Int..

[102] Haertling G.H., Land C.E., Mckinney I.D. (1970). Ferroelectric Ceramic Optical Retardation Devices. U.S. Patent.

[103] Somwan S., Ngamjarurojana A., Limpichaipanit A. (2016). Dielectric, ferroelectric and induced strain behavior of PLZT 9/65/35 ceramics modified by Bi_2_O_3_ and CuO co-doping. Ceram. Int..

[104] Samanta S., Sankaranarayanan V., Sethupathi K. (2018). Effect of Nb and Fe co-doping on microstructure, dielectric response, ferroelectricity and energy storage density of PLZT. J. Mater. Sci. Mater. Electron..

[105] Zhang X., Jin Y., Yao Z., Ye Q., Qu R., Cai H. (2017). Temperature characteristic of electro-optic effect of PMN-PT transparent ceramic. Chin. J. Lasers.

[106] Wang A.-P., Feng M., Wang W., Li H., Zhao X., Xu H., Ke H., Zhou Y. (2018). Influence of composition ratio on ferroelectric, magnetic and magnetoelectric properties of PMN-PT/CFO composite thin films. J. Mater. Sci. Mater. Electron..

[107] Zhang X., Ye Q., Cai H., Qu R. (2014). Polarization-independent electro-optic modulator based on PMNT electrically-controlled birefringence effect and Sagnac interferometer. Opt. Laser Technol..

[108] Qiao L., Ye Q., Gan J., Cai H., Qu R. (2011). Optical characteristics of transparent PMNT ceramic and its application at high speed electro-optic switch. Opt. Commun..

[109] Chen X., Chen R., Chen Z., Chen J., Shung K., Zhou Q. (2016). Transparent lead lanthanum zirconate titanate (PLZT) ceramic fibers for high-frequency ultrasonic transducer applications. Ceram. Int..

[110] Li C., Luo W., Liu X., Xu D., He K. (2016). PMN-PT/PVDF nanocomposite for high output nanogenerator applications. Nanomaterials.

[111] Nikl M., Mihokova E., Mares A., Vedda A., Blazek K. (2000). Traps and timing characteristics of LuAG:Ce^3+^ scintillator. Phys. Status Solidi.

[112] Zhou Z., Li W., Song J., Mei B., Yi G., Yang Y. (2019). Application of Judd-Ofelt theory in analyzing Nd^3+^ doped SrF_2_ and CaF_2_ transparent ceramics. J. Eur. Ceram. Soc..

[113] Wu T., Wang L., He H., Wang H., Shi Y. (2021). Research Progress of Lu_3_Al_5_O_12_-based Scintillation Ceramics. Chin. J. Lumin.

[114] Blasse G. (1994). Scintillator materials. Chem. Mater..

[115] Zhou Z., Li W., Song J., Yi G., Su L. (2017). Synthesis and characterization of Nd^3+^ doped SrF_2_ nanoparticles prepared by precipitation method. Ceram. Int..

[116] Yi G., Li W., Song J., Mei B., Zhou Z., Su L. (2018). Structural, spectroscopic and thermal properties of hot-pressed Nd:(Ca_0.94_ Gd_0.06_)F_2.06_ transparent ceramics. J. Eur. Ceram. Soc..

[117] Chen X., Hu Z., Cao M., Hu C., Liu S., Chen H., Shi Y., Kou H., Xie T., Vedda A. (2018). Influence of cerium doping concentration on the optical properties of Ce,Mg:LuAG scintillation ceramics. J. Eur. Ceram. Soc..

[118] Hostaša J., Cova F., Piancastelli A., Fasoli M., Zanelli C., Vedda A., Biasini V. (2019). Fabrication and luminescence of Ce-doped GGAG transparent ceramics, effect of sintering parameters and additives. Ceram. Int..

[119] Trofimova A., Jacobsohn L. (2020). Radioluminescence of Lu_3_Al_5_O_12_:Ce single crystal and transparent polycrystalline ceramic at high temperatures. Ceram. Int..

[120] Bartosiewicza K., Horiaib T., Yamajib A., Yoshikawab A., Kurosawab S., Yoshinob M., Zorenkoa Y. (2020). Effects of La doping on the crystal growth, phase stability and scintillation properties of Lu_3_Al_5_O_12_ single crystals. Mater. Sci. Eng. B-Adv..

[121] Pagano S., Lombardo G., Costanzi E., Balloni S., Bruscoli S., Flamini S., Coniglio M., Valenti C., Cianetti S., Marinucci L. (2021). Morpho-functional effects of different universal dental adhesives on human gingival fibroblasts: An in vitro study. Odontology.

[122] Abe S., Seitoku E., Iwadera N., Hamba Y., Yamagata S., Akasaka T., Kusaka T., Inoue S., Yawaka Y., Iida J. (2016). Estimation of Biocompatibility of Nano-Sized Ceramic Particles with Osteoblasts, Osteosarcomas and Hepatocytes by Static and Time-Lapse Observation. J. Biomed. Nanotechnol..

[123] Manonmani R. (2021). Novel nano triphasic bioceramic composite coating on 316L SS by electrophoretic deposition process for enhanced corrosion resistance and cell proliferation. J. Aust. Ceram. Soc..

[124] Leyva J.H.R., Vitale G., Hethnawi A., Hassan A., Perez-Zurita M.J., Ruiz-Esparza G.U., Nassar N.N. (2018). Mechanism of hierarchical porosity development in hexagonal boron nitride nanocrystalline microstructures for biomedical and industrial applications. ACS Appl. Nano Mater..

[125] Zhang Z., Zhang Y., Liu D., Zhang Y., Zhao J., Zhang G. (2022). Bubble behavior and its effect on surface integrity in laser induced plasma micro-machining silicon wafer. ASME J. Manuf. Sci. Eng..

[126] Ming W., Shen F., Zhang G., Liu G., Du J., Chen Z. (2021). Green machining: A framework for optimization of cutting parameters to minimize energy consumption and exhaust emissions during electrical discharge machining of Al 6061 and SKD 11. J. Clean. Prod..

[127] Zhang Y., Zhang G., Zhang Z., Zhang Y., Huang Y. (2022). Effect of assisted transverse magnetic field on distortion behavior of thin-walled components in WEDM process. Chin. J. Aeronaut..

[128] Huang H., Zhigilei L.V. (2022). Computational study of laser fragmentation in liquid: Phase explosion, inverse Leidenfrost effect at the nanoscale, and evaporation in a nanobubble. Sci. China Phys. Mech..

[129] Ming W., Zhang S., Zhang G., Du J., Ma J., He W., Cao C., Liu K. (2022). Progress in modeling of electrical discharge machining process. Int. J. Heat Mass Transfer..

[130] Mauroy V., Blanc W., Ude M., Trzesien S., Chaussedent S. (2013). Erbium-doped transparent glass ceramic optical fibres: Characterization using mass spectroscopy and molecular dynamics modeling. Proceedings of the 2012 Photonics Global Conference.

[131] Xu X., Zhao J., Chen X., Xu Q., Wang T., Yu S., Xu X., Qiao S., Du J., Fan X. (2020). Ca^2+^/Sr^2+^/Ba^2+^ dependent phase separation, nanocrystallization and photoluminescence in fluoroaluminosilicate glass. J. Am. Ceram. Soc..

